# Bioactive goldenseal (*Hydrastis canadensis*) fraction suppresses multidrug-resistant *Pseudomonas aeruginosa* in a rat burn wound model: oral and topical administration

**DOI:** 10.1038/s41598-026-60341-z

**Published:** 2026-09-02

**Authors:** Mohamed Samir Turkey, Jilan A. Nazeam, Farag F. Sherbiny, Sulaiman M. Alnasser, Walied Abdo, Tohada M. AL-Noshokaty, Shimaa A. Abass

**Affiliations:** 1 Department of Microbiology and Immunology, Faculty of Pharmacy, October 6 University, Giza, Egypt; 2 Department of Pharmacognosy, Faculty of Pharmacy, October 6 University, Giza, Egypt; 3 Department of Pharmaceutical Organic Chemistry, Faculty of Pharmacy, Al-Azhar University, Cairo, Egypt; 4 Department of Pharmacology and Toxicology, College of Pharmacy, Qassim University, Qassim, 51452 Saudi Arabia; 5 Department of Pathology, Faculty of Veterinary Medicine, Kafrelsheikh University, Kafrelsheikh, Egypt; 6 Biochemistry Department, Faculty of Pharmacy, Heliopolis University, Cairo, 11785 Egypt; 7 Department of Biochemistry, Faculty of Pharmacy, Kafer El-Sheikh University, Kafr El-Sheikh, Egypt

**Keywords:** Goldenseal, *Hydrastis canadensis*, Multidrug-resistant *Pseudomonas aeruginosa*, Burn wound healing, Biofilm inhibition, Molecular docking, Biochemistry, Drug discovery, Microbiology, Plant sciences

## Abstract

Multidrug-resistant (MDR) *Pseudomonas aeruginosa* is a prominent pathogen implicated in burn wound infections, creating substantial therapeutic obstacles due to its antibiotic resistance and enhanced virulence mediated by quorum sensing (QS). This study explored the anti-infective and wound-healing properties of a standardized extract from *Hydrastis canadensis* (Goldenseal; GS) using integrated in silico, in vitro, and in vivo approaches. LC–ESI–MS/MS profiling identified 52 phytochemicals, including alkaloids, flavonoids, phenolic acids, and glycosides, in the extract. Molecular docking against QS regulators (LasR, LasI, RhlR, and RhlI) revealed preferential binding to LasR, with naringenin-7-O-glucoside showing the highest affinity and chlorogenic and orotic acids exhibiting selective interactions. In vitro, GS demonstrated antibacterial activity (MIC = 3.125 mg/mL), significantly inhibited biofilm formation, and reduced *lasR* and *rhlR* expression by 95.3% and 84.8%, respectively (*p* < 0.001). In a rat model of second-degree burns, both oral and topical GS administration was evaluated in vivo, revealing enhanced wound contraction, increased collagen deposition, and improved histopathological outcomes. These outcomes were linked to a reduction in TNF-α and IL-1β levels, alongside an increase in VEGF and bFGF levels, as well as the upregulation of antimicrobial peptides, including CAMP and RBD2. Moreover, the efficacy of topical application surpassed that of oral administration. These findings indicate that GS suppresses QS-regulated virulence and promotes wound repair, supporting its potential application as an adjunctive therapeutic approach for burn wound infections caused by MDR *P. aeruginosa*.

**Supplementary Information:**

The online version contains supplementary material available at https://doi.org/10.1038/s41598-026-60341-z.

## Introduction

Wound and burn injuries represent a substantial global health concern as they compromise the skin’s protective barrier and impair both local and systemic immune functions. Disruption of the barrier function allows microbial invasion and increases the risk of severe infections. Additionally, burn trauma induces intricate inflammatory and immunological responses that can intensify tissue damage and hinder the healing process. Together, these factors contribute to heightened morbidity, emphasizing the importance of developing effective therapeutic strategies to address burn-related complications^1^. Nearly all wounds and burns show increased vulnerability to microbial infections due to a weakened immune response^2^.

Multidrug-resistant (MDR) pathogens constitute an escalating global health challenge, undermining the efficacy of conventional antimicrobial therapies and complicating infection management. *Pseudomonas aeruginosa* is of particular concern owing to its intrinsic resistance mechanisms, extensive repertoire of virulence factors, and remarkable adaptability within clinical environments. This opportunistic microorganism is a leading contributor to serious infections among burn patients and individuals with weakened immune systems, and its ability to persist and resist multiple antibiotics creates significant treatment difficulties. Its adaptive mechanisms enable survival in hostile environments, further complicating the control of infection. Additionally, the emergence of multidrug-resistant strains limits the effectiveness of conventional therapies and necessitates the development of alternative antimicrobial strategies^3^.

The World Health Organization has classified multidrug-resistant *Pseudomonas aeruginosa* as a critical priority pathogen, underscoring the urgent need for novel therapeutic strategies to address this growing concern. Infections caused by this bacterium, such as endocarditis, urinary tract infections, ventilator-associated pneumonia, and chronic wound infections, present significant management challenges. These difficulties often lead to prolonged hospital stays, increased healthcare expenditure, and elevated mortality rates^4,5^. A major determinant of *P. aeruginosa* virulence is its ability to develop biofilms, which are highly organized bacterial communities embedded within a self-produced extracellular matrix containing polysaccharides, proteins and lipids. This biofilm lifestyle markedly increases antimicrobial tolerance by limiting antibiotic diffusion and promoting bacterial persistence in harsh environmental conditions. Biofilms are implicated in most chronic bacterial infections, and cells within these structures are considerably more difficult to eliminate compared to their planktonic (free-living) forms^6,7^.

Given the growing constraints associated with traditional antibiotic treatments, quorum-sensing inhibition has gained recognition as a viable anti-infective strategy. By disrupting the bacterial communication networks that govern biofilm formation and the expression of virulence factors, this method may reduce pathogenicity while exerting minimal selective pressure, thereby potentially curbing the emergence of antimicrobial resistance^8^. The misuse and overuse of antibiotics have accelerated the worldwide spread of multidrug-resistant (MDR) *P. aeruginosa*, particularly in resource-limited environments. This has generated heightened interest in plant-derived antimicrobials, which are abundant in bioactive compounds recognized for their antimicrobial, antibiofilm, and quorum-quenching properties. The safety profiles, accessibility, and cost-effectiveness of natural products render phytochemicals promising candidates for developing novel therapeutics^9^.

Goldenseal (*Hydrastis canadensis* L., Ranunculaceae) has a long history of use in traditional medicine for treating wounds, inflammation, fever, and gastrointestinal disorders^10^. Its antibacterial efficacy has been attributed to its alkaloids, particularly hydrastine and berberine^11^. Goldenseal rhizome and aerial-part fractions exhibit broad-spectrum antibacterial activity against several clinically significant pathogens, including *Staphylococcus aureus*, *Streptococcus mutans*, *S. pyogenes*, *Pseudomonas aeruginosa*, and *Escherichia coli*^12,13^. Importantly, crude fractions frequently demonstrate enhanced antimicrobial and quorum-quenching efficacy relative to purified berberine, suggesting that their bioactivity may arise from synergistic interactions among multiple phytochemical constituents Furthermore, these fractions have shown pronounced activity against methicillin-resistant *S. aureus* (MRSA), with lower MIC values than berberine alone, and have been reported to inhibit ethidium bromide efflux, indicating a potential role in attenuating bacterial resistance^14^.

This study investigated the antibiofilm activity and therapeutic efficacy of goldenseal administered orally and topically against clinical isolates of *P. aeruginosa* in a second-degree burn model. A comprehensive methodological framework integrating in silico molecular docking, in vitro microbiological assays, and in vivo experimental validation was used to rigorously assess efficacy.

## Methods

### Goldenseal purification and fractionation

The powdered roots of Goldenseal (Hydrastis canadensis) were procured from a local Egyptian market. Plant authentication and taxonomic characterization were carried out by Dr. Abdel-Halem Abdel-Mogali, an expert taxonomist affiliated with the Agricultural Research Center, Giza, Egypt. A voucher specimen, coded GS-01, was prepared and deposited in the Herbarium of the Pharmacognosy Department, Faculty of Pharmacy, October 6 University, for future reference. For extraction, 250 g of powdered root material was subjected to aqueous decoction using distilled water at 100 °C for 3 h. The resulting extract was concentrated under reduced pressure using a rotary evaporator until approximately one-tenth of the original volume remained. To eliminate high-molecular-weight constituents, chilled absolute ethanol was added to the concentrated aqueous extract, after which the aqueous filtrate was separated. The filtrate was further concentrated to dryness under vacuum, yielding a purified goldenseal fraction designated GS. The obtained dried fraction was preserved in tightly sealed glass containers at − 20 °C until subsequent analyses.

### Phytochemical characterization of the GS fraction

Metabolite profiling of the purified GS fraction was conducted using an Exion LC AC system coupled to a SCIEX Triple Quad 5500 + mass spectrometer equipped with an electrospray ionization source. The analysis was performed using the multiple reaction monitoring mode. Chromatographic separation was performed on an Ascentis^®^ Express C^18^ column measuring 2.1 × 150 mm, with a particle size of 2.7 μm and pore size of 90 Å. The mobile phase consisted of 5 mM ammonium formate adjusted to pH 8.0 as solvent A, and acetonitrile served as solvent B. Elution was achieved using a linear gradient, increasing solvent B from 5% to 100% over 20 min. The flow rate was maintained at 0.3 mL/min, and the injection volume was 5 µL. Mass detection was performed in the negative ionization mode. The mass acquisition ranges were adjusted to 100–1000 Da for MS¹ and 50–1000 Da for MS², respectively. The ion source parameters were optimized as follows: curtain gas, 25 psi; ion spray voltage, − 4500 V; source temperature, 500 °C; and ion source gases, 45 psi. The declustering potential and collision energy were set to − 80 V and − 35 V, respectively. Metabolites were tentatively assigned using MS-DIAL software version 5. The software is available at: https://systemsomicslab.github.io/compms/msdial/main.html^15^.

### Molecular docking as in-silico study

The binding affinities of the GS fraction compounds toward Pseudomonas aeruginosa quorum-sensing proteins LasR, LasI, RhlR, and RhlI were evaluated using AutoDock Vina software (version 1.2.x; http://vina.scripps.edu). The software utilizes an enhanced scoring function combined with efficient optimization and multithreading algorithms to improve docking precision and computational efficiency, as previously described by Trott and Olson (2010). Crystal structures of LasR (PDB ID: 2UVO) and LasI (PDB ID: 1RO5) were retrieved from the Protein Data Bank (PDB). Due to the lack of experimentally resolved structures for RhlR (UniProt ID: P54292) and RhlI (UniProt ID: P54291) in the PDB database, their three-dimensional structures were generated through homology modeling using the Swiss-Model server based on UniProt amino acid sequences. Structural quality and reliability of the generated models were validated using Ramachandran plot assessment and ProSA-Z score analysis^16^.

The two-dimensional chemical structures of the investigated compounds and co-crystallized ligands were generated using ChemBioDraw Ultra 14.0. Validation of the docking protocol was achieved through re-docking of the co-crystallized ligands, resulting in a root mean square deviation (RMSD) value of 0.9 Å, thereby confirming the reliability and reproducibility of the docking settings employed in the current study.

To further investigate ligand–protein interactions and minimize false-positive and false-negative docking predictions, molecular dynamics (MD) simulations were subsequently performed to identify the most energetically favorable binding conformations. MD simulations were carried out for 400 ps at 300 K using a simulation time step of 2.0 fs and a non-bonded interaction cut-off distance of 10 Å. Temperature and pressure parameters were maintained constant throughout the simulation period. Additionally, all backbone atoms within the complexes were restrained to their initial coordinates using a harmonic force constant of 2.0 kcal/(mol·Å²). Docking poses, molecular interactions, and structural visualizations were analyzed using PyMOL Molecular Graphics System (version 2.0). The software is available at: https://pymol.org/^17^.

### In vitro antimicrobial effect of GS fraction

#### Bacterial isolates and ethical approval

A total of 100 clinical isolates of *Pseudomonas aeruginosa* were collected from wound and burn infections across several healthcare institutions in Egypt, with the majority obtained from October 6 University Hospital, during the period from June to September 2025. Ethical clearance for the use of these isolates was granted by the Faculty of Pharmacy, October 6 University (approval no. PRE-Ph-2508002). The isolates were retrieved from the microbiology laboratory records, and all samples were anonymized prior to analysis. Written informed consent was obtained as part of the standard clinical practice at the time of specimen collection.

The initial identification of the isolates was performed using conventional microbiological techniques and subsequently confirmed using the VITEK 2 Compact system. One representative isolate, *P. aeruginosa* (C1), was deposited in the Culture Collection of Ain Shams University under accession number CCASU-2025-81 (WDCM). Further molecular confirmation was performed through 16 S rRNA gene sequencing, and the resulting sequence was submitted to the GenBank database. (accession number PV640469)^3^. https://www.ncbi.nlm.nih.gov/nuccore/PV640469.1/.

### Phenotypic detection of biofilm formation

#### Evaluation of biofilm formation

The biofilm-forming capacity of clinical *P. aeruginosa* isolates was quantitatively evaluated using a modified 96-well microtiter plate method. Overnight cultures were prepared in Luria–Bertani (LB) broth at 37 °C, adjusted to 0.5 McFarland turbidity, and subsequently diluted 1:100 with fresh LB medium. Aliquots of 200 µL from each bacterial suspension were transferred into sterile flat-bottom 96-well plates, with each isolate tested in triplicates. Sterile LB broth was used as the negative control, and *P. aeruginosa* ATCC 12,924 was used as the positive control. The plates were incubated statically at 37 °C for 24 h. Following incubation, planktonic cells were carefully discarded, and the wells were gently washed with phosphate-buffered saline to remove non-adherent cells. The remaining adherent biofilm biomass was fixed with 99% methanol for 15 min, air-dried, and stained with 2% Hucker’s crystal violet for 15 min. After removing the excess stain by rinsing, the retained dye was dissolved using 33% acetic acid. Absorbance was measured at 595 nm using a TECAN microplate reader. The optical density cutoff value was calculated as the mean absorbance of the negative control plus three standard deviations. According to the obtained OD values, isolates were classified as non-biofilm producers when OD ≤ ODc, weak biofilm producers when ODc < OD ≤ 2 × ODc, moderate biofilm producers when 2 × ODc < OD ≤ 4 × ODc, and strong biofilm producers when OD > 4 × ODc^18^.

### Verification of non-growth-inhibitory effect sub-MIC concentration by viable count assay

To verify that the selected sub-inhibitory concentration (1/2 MIC) of the goldenseal fraction did not significantly affect the growth of *P. aeruginosa*, a viable count assay was performed under the same experimental conditions used in the antibiofilm assay. A standardized bacterial suspension of *P*. *aeruginosa* was prepared in sterile broth and adjusted to approximately 1 × 10⁶ CFU/mL. The aliquots were incubated either without treatment, serving as the untreated control, or with the goldenseal fraction at half the minimum inhibitory concentration (MIC) at 37 °C for 24 h. Following incubation, the samples underwent serial dilution with sterile normal saline, were plated onto nutrient agar, and subsequently incubated again at 37 °C for 18–24 h. The number of viable colonies was estimated, and the results were expressed as log₁₀ CFU/mL. The assay was performed in triplicate, and the mean viable counts ± standard deviation (SD) were determined. The absence of a statistically significant reduction in viable counts relative to the untreated control was considered indicative of a non-growth-inhibitory sub-MIC^19^.

### Molecular identification and functional characterization

The clinical isolate P1 was selected for molecular identification because it exhibited the highest antimicrobial resistance pattern and the most pronounced biofilm-forming capacity. Identification was performed using 16S rRNA gene sequencing. Following overnight growth in nutrient broth, the bacterial biomass was collected by centrifugation. Genomic DNA was extracted using the GeneJET™ PCR Purification Kit (Thermo Scientific) following the manufacturer’s protocol. The quality of the extracted DNA was checked by electrophoretic separation on a 1% agarose gel, after which the DNA samples were preserved at − 20 °C until use. The 16S rRNA gene was amplified using the universal bacterial primer pair 27 F, 5′-AGAGTTTGATCCTGGCTCAG-3′, and 1492R, 5′-TACGGTTACCTTGTTACGACTT-3′.’ PCR reactions were prepared in a final volume of 25 µL containing Maxima^®^ Hot Start PCR Master Mix, 0.4 µL of each primer at a concentration of 10 µM, 5 µL of template DNA, and nuclease-free water to complete the reaction volume. A no-template control was included in each run to verify that the reaction mixture was free of contamination. The resulting PCR product was sequenced, and the obtained nucleotide sequence was analyzed using BLASTn against the NCBI GenBank database. After confirmation, the 16 S rRNA gene sequence of the selected isolate was deposited in GenBank^20^.

### Antibacterial screening of GS fraction

The antibacterial potential of the GS aqueous fraction was evaluated using an agar well diffusion assay. Standardized suspensions of clinical *P. aeruginosa* isolates, adjusted to 0.5 McFarland turbidity, were inoculated uniformly onto Mueller–Hinton agar plates. Wells were aseptically punched into the agar surface and loaded with 200 µL of the GS aqueous fraction at a concentration of 100 mg/mL. The plates were then incubated at 37 °C for 24 h. After incubation, antibacterial activity was assessed by measuring the diameter of the clear inhibition zones formed around each well, and the recorded values were used to estimate the inhibitory effect of the GS fraction^9^.

### Evaluation of MIC

The antibacterial potency of the GS fraction was evaluated by determining its minimum inhibitory concentration using the broth microdilution technique, in accordance with the 2022 CLSI recommendations. Three multidrug-resistant *P. aeruginosa* isolates, designated P1, P2, and P3, were selected for testing based on their strong biofilm-forming capacities. The GS fraction was prepared at an initial concentration of 100 mg/mL and subjected to two-fold serial dilution to obtain final concentrations ranging from 50 to 0.049 mg/mL. The assay was done in sterile flat-bottomed 96-well microtiter plates. Each well received 100 µL of the corresponding GS dilution and 100 µL of a bacterial suspension prepared in Mueller–Hinton broth. The plates were then incubated at 37 °C for 24 h. The MIC was defined as the lowest concentration of the GS fraction that prevented visible bacterial growth. All assays were performed in triplicates.

### Assessment of sub-inhibitory effects on biofilm formation

To investigate the antibiofilm efficacy of the goldenseal fraction at sub-inhibitory concentrations, a microtiter plate biofilm assay was performed. Each isolate was treated with the aqueous fraction at half the minimum inhibitory concentration (MIC), and untreated wells served as controls. Following a 24-hour incubation period, the inhibition of biofilm formation was determined using the following equation:

$$Biofilm~Inhibition~\left( \% \right){\text{ }} = \:\frac{{OD\:control - OD\:sample}}{{OD\:control}} \times 100$$

### Molecular screening of quorum-sensing genes in *P. aeruginosa* by PCR

Genomic DNA was extracted from *P. aeruginosa* isolates using the QIAamp DNA Mini Kit (Qiagen, Cat. No. 51304) according to the manufacturer’s instructions. Briefly, bacterial samples were lysed with protease and Buffer AL, incubated at 56 °C for 10 min, mixed with 96% ethanol, and loaded onto the spin columns. After washing with AW1 and AW2 buffers, DNA was eluted in 100 µL Buffer AE. The quorum-sensing genes *lasI*, *lasR*, *rhlI*, and *rhlR* were amplified by PCR in a 25 µL reaction containing EmeraldAmp GT PCR Master Mix, gene-specific primers, template DNA, and nuclease-free water. Amplification was performed with an initial denaturation at 94 °C for 5 min, followed by 35 cycles of denaturation, annealing at 55–60 °C, and extension at 72 °C, with a final extension at 72 °C. PCR products were separated on a 1.5% agarose gel in 1× TBE buffer, stained with ethidium bromide, and visualized under ultraviolet (UV) light. A 100 bp DNA ladder was used as the molecular size marker. This assay was used to detect quorum-sensing genes in multidrug-resistant *P. aeruginosa* isolates^21–24^.

### Transcriptional analysis

Three clinical *P. aeruginosa* isolates, designated P1, P2, and P3, were selected for transcriptional analysis based on their robust biofilm-forming phenotypes and pronounced antimicrobial resistance profiles. Each isolate was initially subcultured on Luria–Bertani (LB) agar plates and incubated at 37 °C for 24 h. A single well-isolated colony from each culture was inoculated into 10 mL of LB broth and incubated overnight at 37 °C under agitation at 225 rpm. Subsequently, overnight cultures were diluted 1:100 in fresh LB broth and assigned to either untreated control conditions or treatment with sub-MIC of the goldenseal fraction. The cultures were incubated under identical conditions for 6–8 h, corresponding to the late exponential to early stationary growth phases. Thereafter, 10 mL aliquots from each culture were harvested by centrifugation at 5000 rpm for 5 min at 4 °C. The supernatants were carefully removed, and the resulting bacterial pellets were either immediately stored at − 80 °C or processed directly for RNA extraction^3^.

### Total RNA extraction and qRT-PCR preparation

Total RNA was extracted from *P. aeruginosa* isolates using the RNeasy Mini Kit (Qiagen, Cat. (No. 74104) with minor modifications. The cultures were stabilized with RNAprotect Bacteria Reagent, centrifuged, treated with TE-lysozyme buffer, and lysed using Buffer RLT and ethanol. RNA was purified using RNeasy spin columns, washed with RW1 and RPE buffers, treated with on-column DNase, and eluted in RNase-free water. qRT-PCR was performed using the QuantiTect SYBR Green PCR Kit (Qiagen, Cat. No. 204141). Each 25 µL reaction contained SYBR Green Master Mix, reverse transcriptase, gene-specific primers, RNase-free water, and RNA templates. The primer sequences are listed in Table S1^21,22,25,26^.

### Quantitative real-time PCR (qRT-PCR)

Relative transcription levels of biofilm- and virulence-associated genes were determined using qRT-PCR with a Stratagene MX3005P thermocycler. The reaction conditions comprised reverse transcription at 50 °C for 30 min, followed by an initial denaturation step at 94 °C for 15 min. Amplification was then performed for 40 cycles, including denaturation at 94 °C for 15 s, primer-specific annealing for 30 s, and extension at 72 °C for 40 s. The annealing temperatures were set at 50 °C for 16 S rDNA, 56 °C for *lasI*, 55 °C for *lasR*, 58 °C for *rhlI*, and 60 °C for *rhlR* genes. The specificity of the amplification was confirmed using melting curve analysis. Gene expression levels were normalized against 16 S rDNA and quantified using the 2^−ΔΔCt method^21,22,25,26^.

### In vivo experimental analysis

#### In vivo experimental design and animal ethics

Twenty-four male albino rats weighing 150–180 g and aged 8–10 weeks were supplied by the Egyptian Organization for Biological Products and Vaccines, Giza, Egypt. Animals were housed under standard laboratory conditions at 23 ± 2 °C with a 12 h light/dark cycle and were provided with free access to a standard diet and water. The rats were acclimatized for one week before the start of the experiment. The study protocol was approved by the Institutional Animal Care and Use Committee (IACUC), Kafrelsheikh University, Egypt (approval number KFS-IACUC/359/2025). All experimental procedures complied with institutional regulations, the ARRIVE guidelines, and internationally accepted principles for the ethical care and use of laboratory animals.

### Burn wound induction, anesthesia, and treatment

Animals were anesthetized using ketamine (80 mg/kg) and xylazine (10 mg/kg) administered intraperitoneally prior to burn injury induction. Adequate anesthesia was confirmed by the absence of pedal withdrawal reflexes in the rats. A second-degree burn wound was induced by applying a pre-heated metal bar (2 × 2 cm, 90 °C) to the shaved dorsal skin for 12 s, as previously described^27,28^. Burn wounds were infected with *P. aeruginosa* using sterile cotton swabs. To establish an infected burn wound model, a standardized bacterial inoculum of *P. aeruginosa* was prepared from an overnight culture adjusted to approximately 1 × 10^6^ CFU/mL. A defined volume (100 µL) of the bacterial suspension was carefully applied topically to each burn wound immediately after injury induction. The inoculum was consistently distributed across the wound surface using a sterile swab to ensure a consistent infection.

The animals were randomly divided into four groups (*n* = 6): control, burn, topical GS gel (MIC = 3.12 mg/mL), and oral GS (300 mg/kg suspended in 1% CMC). The treatments were administered daily for 10 days. The oral dose was selected based on a previously published in vivo study to achieve effective tissue concentrations and overcome the first-pass effect^29^. The topical GS formulation was developed using a conventional aqueous gel base containing carboxymethyl cellulose (CMC, 1% w/v) as the gelling agent. CMC was selected because of its biocompatibility, ease of application, and negligible impact on wound healing. The GS fraction was then incorporated into the gel at the desired concentration (MIC: 3.12 mg/mL) with continuous stirring to achieve homogeneous dispersion^30^. After concluding the experiment, the animals were placed under deep anesthesia, and full-thickness skin samples were collected for histological, molecular, and biochemical examinations.

### Measurement of infected burned wound area

Wound progression was assessed by photographing the burn sites at certain time points. Images were captured using a digital camera on days 0, 2, 4, 8, and 12. The infected burn areas were measured using ImageJ software (version 2.0, NIH, USA). The software is available at: https://imagej.nih.gov/ij/. At each time point, the wound size was compared to the original wound area on day 0, and the wound contraction percentage was determined using the following formula: % wound contraction = (current wound area/wound area on day 0) × 100^31^.

### Enzyme-linked immunosorbent assay (ELISA)

The levels of tumor necrosis factor-alpha (TNF-α) and interleukin-1β (IL-1β) in skin tissue homogenates were quantified using enzyme-linked immunosorbent assay (ELISA) kits, according to the manufacturer’s instructions. IL-1β was measured using a commercial kit from Wuhan Boster Biological Technology Ltd., Wuhan, China (Cat. No. EK0393), while TNF-α was determined using an Elabscience Biotechnology Co., Ltd. kit (Cat. No. E-EL-R0019c).

### Hydroxyproline determination assay

Hydroxyproline content was determined according to the method described by Qiu et al. (2014)^32^. Briefly, 1 g of the wound area was homogenized, followed by hydrolysis in 12 N HCl at 120 °C for 3 h. After hydrolysis, the samples were mixed with 3 ml of toluene and agitated for 20 min to remove interferences. The mixture was then centrifuged at 3,000 × g at 20 °C for 10 min. Subsequently, the hydroxyproline in the samples was oxidized using chloramine-T, followed by a reaction with p-dimethylaminobenzaldehyde (DMAB). The hydroxyproline amount was determined spectrophotometrically at 560 nm against a hydroxyproline standard purchased from Sigma-Aldrich, USA (H544909, CAS: 51-35−4).

### Quantitative RT-PCR assessment of wound-healing-related gene expression

Quantitative real-time PCR was performed to assess the expression of *VEGF*, *bFGF*, *CAMP*, and β-defensin 2 in skin tissue samples. Total RNA was extracted using the miRNeasy kit according to the manufacturer’s protocol and reverse-transcribed into cDNA using a Qiagen reverse-transcription kit. Quantitative PCR was conducted using cDNA, SYBR Green master mix, and gene-specific primers, following this protocol: an initial denaturation at 95 °C for 5 min, followed by 40 cycles of 94 °C for 20 s and 60 °C for 1 min. Gene expression levels were quantified using the 2^−ΔΔCt method, normalized to β-actin, with primer details provided in Table S2. Primer specificity and efficiency were confirmed using standard curves generated from serial cDNA dilutions. All primer sets exhibited amplification efficiencies between 90% and 110%, with R² values of ≥ 0.99. Melt curve analysis revealed single specific peaks without primer-dimer formation, while β-actin Ct values remained stable across all experimental groups.

### Histological assessments

Burn wound tissues were preserved in 10% neutral buffered formalin (pH 7.4) prior to histological processing. Following fixation, the specimens were rinsed, passed through ascending concentrations of alcohol for dehydration, and subsequently embedded in paraffin blocks. Sections of 5 μm thickness were prepared from the paraffinized tissues, stained with hematoxylin and eosin (H&E), and examined under a light microscope for histopathological assessment. Quantitative evaluation of wound repair was performed according to the scoring criteria reported by Atiba^27^, which considered inflammatory cell infiltration, granulation tissue development, and re-epithelialization. Each criterion was graded on a scale ranging from 0 to 4, and the total healing score was determined by summing the three individual scores, yielding a maximum possible value of 12.

### Statistical analysis

Statistical analyses were performed using GraphPad Prism version 8.0 and SPSS, and graphs were generated using GraphPad Prism. Data are reported as mean ± SD from three separate experiments.

Intergroup comparisons were performed using one-way analysis of variance (ANOVA), followed by Tukey’s HSD multiple-comparison test. A p-value below 0.05 was considered statistically significant.

## Results

### Chemical characterization of GS fraction

Extraction and purification of the plant material yielded 70 g of purified GS fraction from 1 kg of powdered starting material, corresponding to a 7% (w/w) yield. The chemical composition of the goldenseal fraction was characterized using liquid chromatography–electrospray ionization tandem mass spectrometry (LC–ESI–MS/MS). Analysis in multiple reaction monitoring (MRM) mode enabled the detection of a broad range of phenolic and flavonoid compounds. Six major constituents—chlorogenic acid, *p*-coumaric acid, gallic acid, 3,4-dihydroxybenzoic acid, ferulic acid, and syringic acid—were identified based on their precursor ions (Q1), product ions (Q3), retention times, and comparisons with reference spectra (Table S3; Fig. S1, Supplementary File). In total, 52 metabolites were detected, encompassing diverse chemical classes, including organic acids, amino acids, phenolic acids, phenols, flavonoids, nucleosides, alkaloids, sugars, coumarins, and terpenes. This study is the first to provide a comprehensive LC–ESI–MS/MS-MRM profiling of metabolites in goldenseal root (Table S4-S5, Supplementary file).

### Molecular docking study

To identify potential binding affinities, chemical compounds derived from the GS fraction were docked with several key regulatory proteins involved in the quorum sensing (QS) network. To assess the binding affinities, compounds identified from the GS fraction were docked against key quorum sensing (QS) regulatory proteins (*LasR*,* LasI*,* RhlR*,* and RhlI*)^33^. Docking analysis of 52 representative phytochemicals revealed significant interactions with these receptors, with the binding energies summarized in Table S6, Supplementary file. Several compounds formed hydrogen bonds, hydrophobic contacts, and aromatic stacking interactions with critical amino acid residues, suggesting their potential as scaffold inhibitors of both quorum sensing and subsequently inhibits biofilm assembly.

The results of the docking study revealed that goldenseal-derived compounds exhibited notable binding affinities for the active site of the *LasR* receptor, with scores ranging from − 2.12 to − 9.38 kcal/mol. Among all tested compounds, naringenin-7-O-glucoside (compound 29) demonstrated the highest binding affinity at − 9.38 kcal/mol. This suggests that compound 29 may be the most promising inhibitor for *LasR*, exhibiting strong potential for stable and favorable molecular interactions. It outperformed all other candidates, including compound 21 (–8.09 kcal/mol) and compounds 13, 20, and 33, which showed comparable binding scores in the range of − 7.96 to − 7.98 kcal/mol.

Naringenin-7-O-glucoside (compound 29) exhibited a binding mechanism comparable to that of the co-crystallized ligand OHN, involving a combination of hydrophilic and hydrophobic interactions that enhanced its affinity for the target protein’s binding site. The 4-hydroxyphenyl group formed a hydrogen bonding interaction with the backbone carbonyl group of Val76. Additionally, the hydroxyl group on the chromene nucleus established a hydrogen bonding interaction with Arg61, while the oxygen atom at the 7-position of the chromene ring formed a hydrogen bonding interaction with Tyr56. Moreover, the two hydroxyl groups on the pyran moiety engaged in hydrogen bonding with Trp60 and formed a water-mediated interaction with Arg61. The chromene ring was further stabilized through aromatic stacking interactions with Tyr56 and Tyr64 and was positioned within a hydrophobic pocket formed by Leu36, Ile52, Ala70, and Val76.

**Fig. 1:**
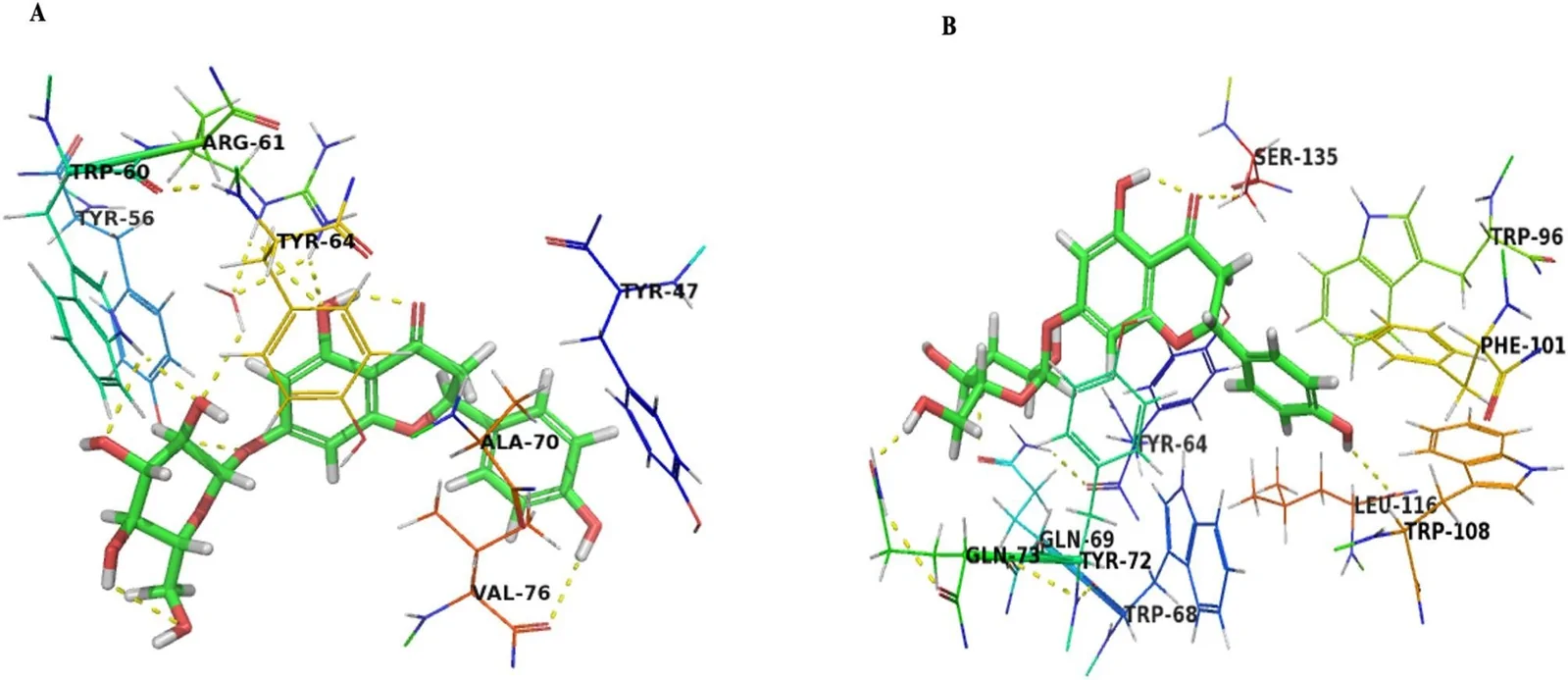
(**A**): Predicted binding mode of compound 29 with the *LasR* binding site, (**B**): Predicted binding mode of compound 29 with the *RhIR* binding site. Interactions between H-bonded atoms are indicated by yellow dotted lines. Hydrogen (white), nitrogen (blue), oxygen (red) and sulfur (yellow).

The 4-hydroxyphenyl moiety participated in aromatic stacking interactions with Tyr47 and formed hydrophobic contacts with Leu40, Ala50, Cys79, Thr80, and Leu125. Additionally, the pyran moiety was stabilized by hydrophobic interactions with Thr75, Trp88, Ile92, Tyr93, Phe101, Ala105, and Leu110, which collectively enhanced the binding affinity of compound 29 for the *LasR* binding site (Fig. 1A). Naringenin has been shown to impedes quorum sensing in *P. aeruginosa* by interfering with LasR-mediated signaling pathways, leading to a decrease in biofilm formation and a reduction in the synthesis of virulence factors^34^. In addition, flavonoids are well documented as quorum-sensing inhibitors and antibiofilm agents against gram-negative bacteria^35^. These findings support the plausibility of the observed docking affinity and suggest that naringenin-7-O-glucoside may exert its effects either directly or following metabolic conversion to its aglycone form.

Among the compounds screened against the *LasI* receptor, orotic acid (compound 38) demonstrated the highest binding affinity, with a value of − 7.42 kcal/mol. This indicates that compound 38 likely forms the most stable and favorable interactions with the *LasI* active site. The next most promising candidate, compound 52, exhibited a binding affinity of − 7.06 kcal/mol, followed by compounds 27, 5, and 2, which showed slightly lower affinities.

**Fig. 2:**
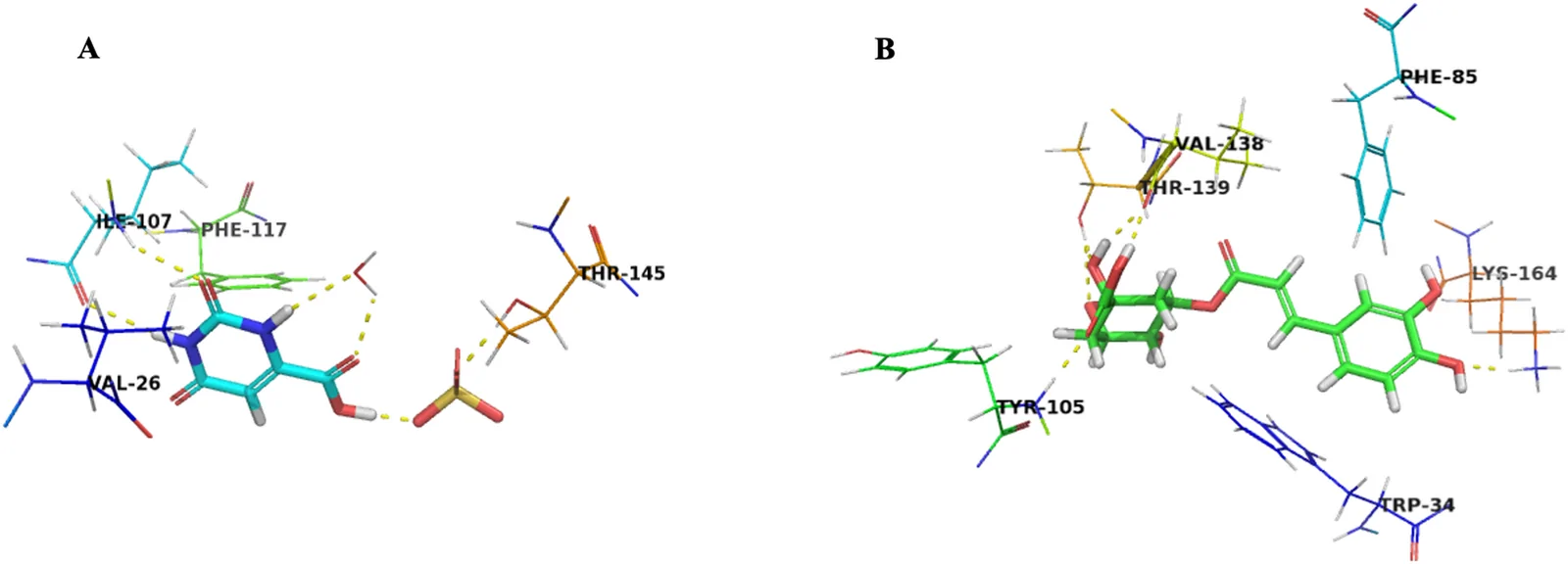
(**A**): Predicted binding mode of compound 38 with the *LasI* binding site. (**B**): Predicted binding mode of compou**nd 27 w**ith the *Rh1I* binding site. Interactions between H-bonded atoms are indicated by yellow dotted lines. Hydrogen (white), nitrogen (blue), oxygen (red) and sulfur (yellow).

Molecular docking analysis of orotic acid (compound 38) revealed that its pyrimidine scaffold was accommodated within the LasI binding pocket through multiple stabilizing interactions. The N–H group at position 1 of the pyrimidine ring formed a hydrogen bond with the backbone carbonyl of Ile107, whereas the carbonyl group at position 2 interacted with the backbone amide of the same residue. Additional water-mediated hydrogen bonds were observed between the polar functional groups of the pyrimidine core/carboxylate region and the backbone amide groups of Gly147 and Thr145 in the active site. Moreover, the pyrimidine ring exhibited π-stacking interactions with Phe117 and contributed to hydrophobic contacts with Val26, Ile107, and Val148. These combined water-bridged, aromatic, and hydrophobic interactions may contribute to the molecular recognition of orotic acid at the receptor site. However, its relatively weak binding affinity toward LasI could be explained by the suboptimal placement of its highly polar pyrimidine nucleus within a predominantly hydrophobic pocket defined by surrounding residues (Fig. 2A).

For the *RhIR* receptor, both compound 29 (naringenin-7-O-glucoside) and compound 45 (Daidzein) exhibited the strongest binding affinity, each with a value of − 8.47 kcal/mol, suggesting that these two compounds are equally potent in terms of interaction strength and binding stability with *RhIR*. Following them, compound 27 also demonstrated strong affinity at − 7.85 kcal/mol, while compound 4 showed a comparable binding score of − 7.76 kcal/mol. Compounds 21 and 24 displayed slightly lower but equal affinities, each with a binding energy of − 7.66 kcal/mol.

The binding mode of compound 29 with the *RhIR* receptor revealed several key interactions. The 4-hydroxyphenyl group formed a hydrogen bonding interaction with the backbone carbonyl group of Leu116, while the carbonyl group of the chromene nucleus established a hydrogen bonding interaction with Ser135. Additionally, the hydroxy and hydroxymethyl groups of the pyran moiety engaged in hydrogen bonding interactions with Gln69 and Gln73, respectively. The chromene moiety was further stabilized by aromatic stacking interactions with Tyr43, Tyr64, and Tyr68 and was positioned within a hydrophobic pocket formed by Ala44, Cys46, Met60, and Ile84. The 4-hydroxyphenyl moiety participated in aromatic stacking interactions with Trp68, Trp96, Phe101, and Trp108 and formed hydrophobic interactions with Thr83, Ala111, Leu107, Leu116, and Ala137. Additionally, the pyran moiety was stabilized by hydrophobic interactions with Met60 and the nonpolar region of Gln69 (Fig. 1B).

With a binding affinity of − 7.98 kcal/mol, compound 27 (Chlorogenic acid) exhibited the highest affinity among all compounds tested against the *Rh1I* receptor, making it the most promising candidate for robust and stable ligand–receptor interactions. Close contenders included compound 38 (–7.51 kcal/mol), compound 45 (–7.38 kcal/mol), compound 29 (–7.30 kcal/mol), and compound 26 (–7.28 kcal/mol), all of which also demonstrated favorable binding with *Rh1I*.

Docking results for compound 27 indicated that the hydroxyphenyl group was involved in a hydrogen bonding interaction with Lys164, while the dihydroxycyclohexane moiety formed hydrogen bonds with Thr139 and the backbone carbonyl group of Val138. The carboxyl moiety further contributed to binding stabilization through hydrogen bonds with the backbone carbonyl group of Val138 and the backbone amino group of Tyr105. The phenyl moiety was positioned within a hydrophobic pocket formed by Val36, Leu80, Val163, and Leu168, and was further stabilized by aromatic stacking interactions with Trp34 and Phe85. Additionally, the cyclohexane moiety occupied a hydrophobic pocket formed by Leu102, Tyr105, Ala137, Thr140, Met143, and Phe147 (Fig. 2B).

Based on the analysis of the binding affinity values for each fractionated compound, the receptors can be ranked according to the strength of their individual binding interactions. Among the four receptors evaluated (LasR, LasI, RhIR, and Rh1I), *LasR* consistently demonstrated the strongest binding affinities across the majority of the 42 tested compounds. This suggests that *LasR* is the most broadly responsive and potent binding partner, exhibiting the most favorable interactions overall. Notably, compound 29 achieved the highest binding affinity with *LasR*, recording a value of − 9.38 kcal/mol (Table S7, Supplementary file).

In contrast, *RhIR and Rh1I* exhibited strong binding affinities for a few individual compounds; however, their overall performance was less consistent. For *Rh1I*, compound 27 showed the highest binding affinity at − 7.98 kcal/mol, while for *RhIR*, compounds 29 and 45 both exhibited the strongest binding affinities, each with a value of − 8.47 kcal/mol. The weakest overall performance was observed with the *LasI* receptor, which showed the highest affinity in only one case compound 38, with a binding energy of − 7.42 kcal/mol.

**Fig. 3:**
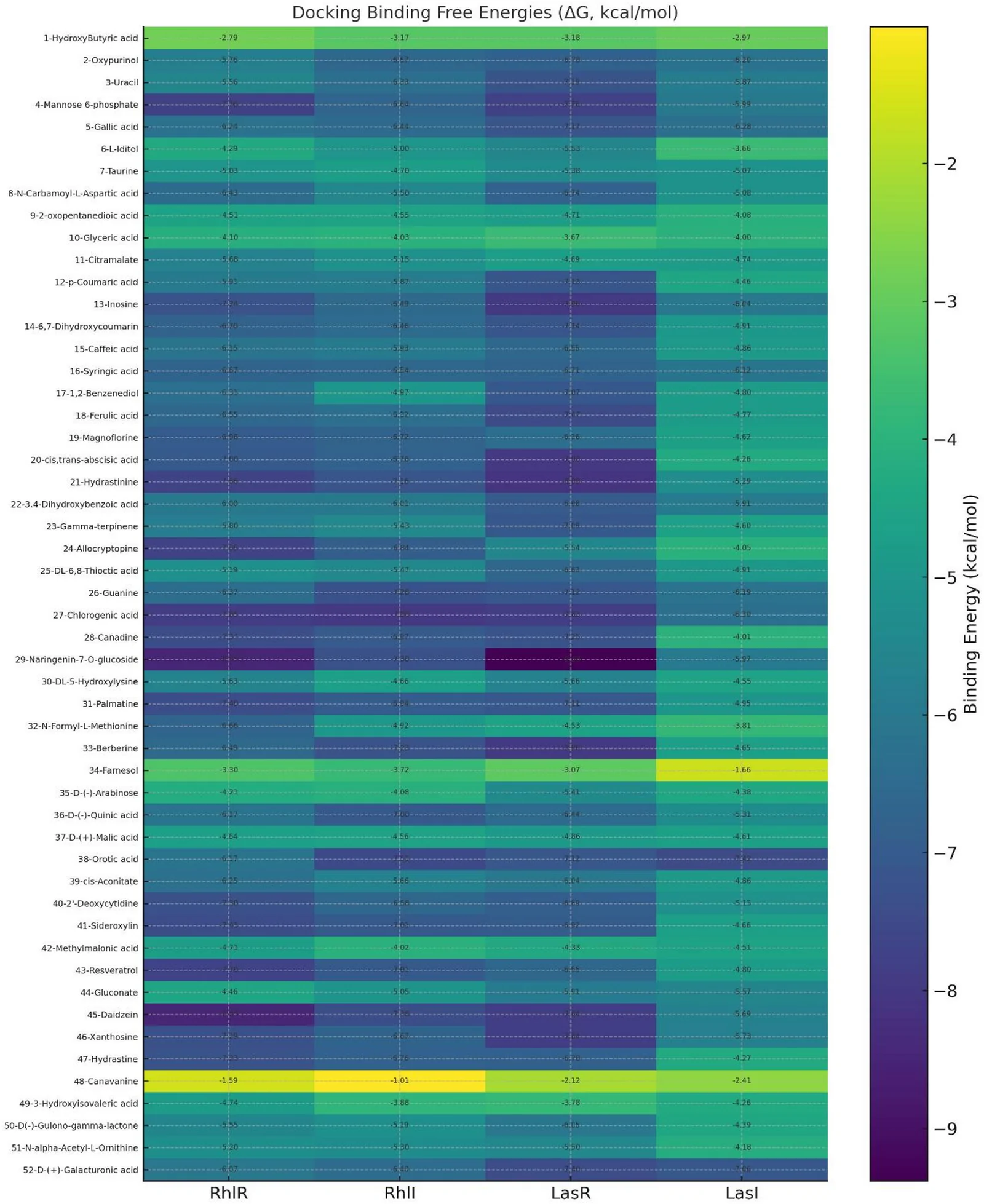
Docking heatmap of *Goldenseal* phytochemicals with quorum-sensing receptors (*RhlR*,* RhlI*,* LasR*, *LasI).* More negative binding free energies (ΔG, kcal/mol) indicate stronger affinities. Naringenin-7-O-glucoside, hydrastinine, berberine, daidzein, and chlorogenic acid showed the strongest interactions, particularly with *LasR*, highlighting its role as the primary molecular target.

The heat map of the docking analysis revealed the distinct binding affinities of goldenseal phytochemicals toward quorum-sensing regulators. *LasR* emerged as the most responsive receptor, with naringenin-7-O-glucoside exhibiting the strongest binding (–9.38 kcal/mol), followed by hydrastinine (–8.09 kcal/mol), berberine (–7.96 kcal/mol), inosine (–7.96 kcal/mol), and daidzein (–7.84 kcal/mol). Chlorogenic acid showed consistent strong affinities across targets (–7.98 kcal/mol for RhlI and − 7.83 kcal/mol for *LasR*), whereas orotic acid demonstrated notable binding to RhlI (–7.51 kcal/mol) and *LasI* (–7.42 kcal/mol). Additional bioactive alkaloids, such as canadine (–7.25 kcal/mol) and palmatine (–7.11 kcal/mol), further supported this binding trend. In contrast, low-molecular-weight metabolites, including canavanine (–2.12 kcal/mol) and farnesol (–3.07 kcal/mol), exhibited minimal interactions (Fig. 3). These results indicate that *LasR* is the most promising molecular target for goldenseal phytochemicals, with several compounds displaying binding free energies below − 7.0 kcal/mol, consistent with a strong anti-virulence potential.

**Fig. 4:**
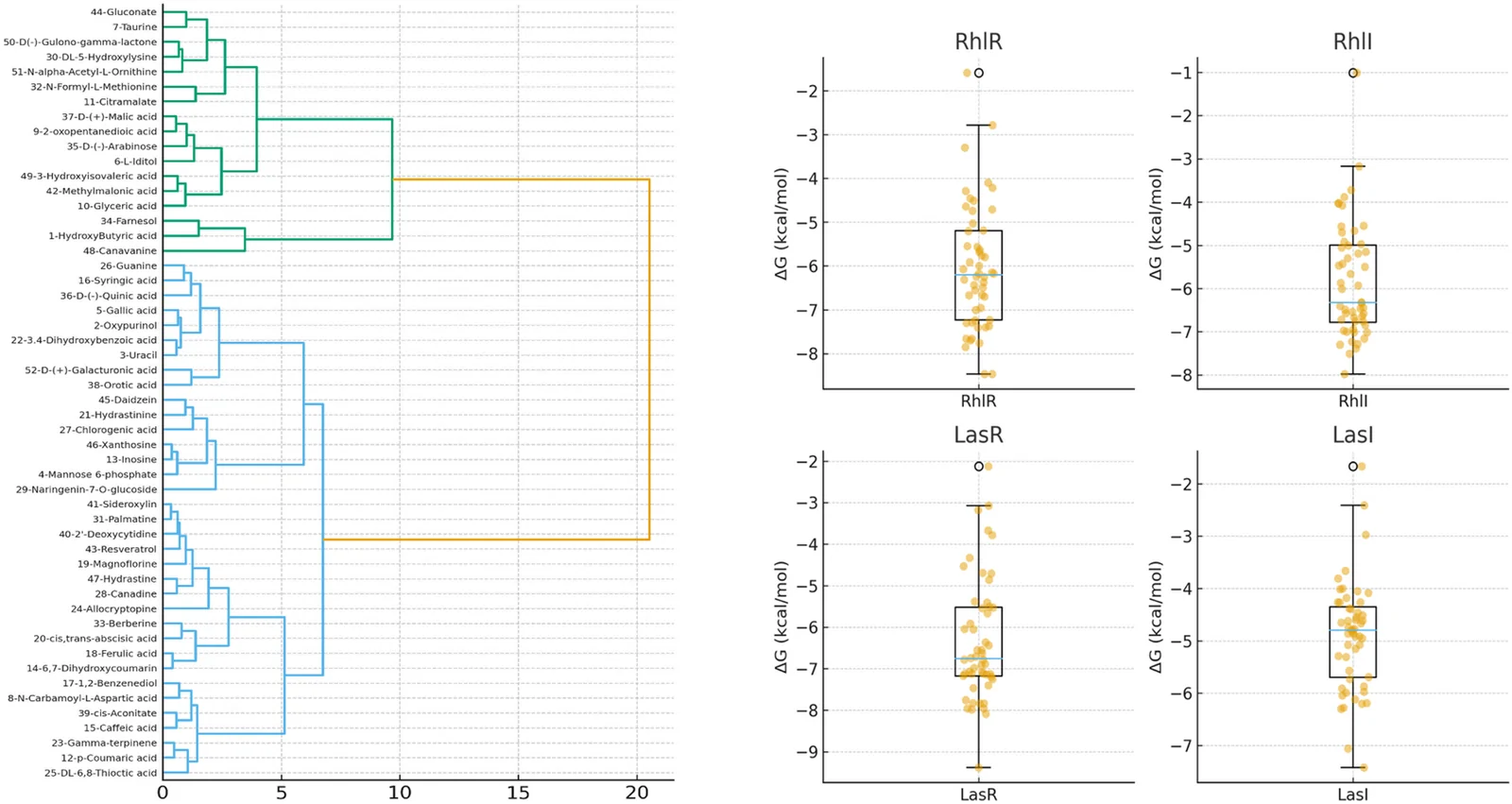
Hierarchical clustering and docking energy distributions of *Goldenseal* phytochemicals. (**A**) Hierarchical clustering dendrogram of 52 identified ligands based on their docking free energy profiles across quorum-sensing receptors (*RhlR*,* RhlI*,* LasR*, and *LasI*). Clustering was performed using Ward’s linkage on Euclidean distances, grouping compounds with similar multi-receptor binding patterns. (**B**) Distribution of docking free energies (ΔG, kcal/mol) for each receptor. Boxplots illustrate median and interquartile ranges, while jittered points represent individual ligand affinities. *LasR* exhibited the most negative (strongest) median ΔG values, indicating higher responsiveness compared to other receptors.

Hierarchical clustering of docking profiles (Fig. 4A) grouped the 52 goldenseal phytochemicals into distinct clusters, reflecting the shared multi-receptor binding patterns. Compounds such as naringenin-7-O-glucoside, chlorogenic acid, berberine, and hydrastinine clustered together, consistent with their strong and broad-spectrum interactions with QS regulators. Distribution analysis of the docking energies (Fig. 4B) further demonstrated that *LasR* exhibited the most negative median ΔG values, confirming it as the most responsive of the three receptors. These findings indicate that goldenseal-derived ligands not only share conserved binding motifs but also preferentially target *LasR*, a key regulator of quorum sensing and biofilm formation.

### Antimicrobial susceptibility testing (AST)

Antimicrobial susceptibility profiling demonstrated pronounced resistance among the isolates to ticarcillin and ticarcillin/clavulanate, as well as piperacillin and piperacillin/tazobactam, with MICs ≥ 128 µg/mL. Likewise, high resistance levels were detected for ceftazidime, amikacin, and cefepime, each showing MICs ≥ 64 µg/mL. Conversely, the isolates exhibited notably lower resistance to colistin. All susceptibility assessments were conducted in accordance with the Clinical and Laboratory Standards Institute (CLSI) guidelines, 2022, as summarized in Table S8 (Supplementary File).

### Biofilm production

All 100 P. aeruginosa isolates demonstrated the capacity to form biofilms. Among these, 60 were categorized as strong biofilm producers, 20 exhibited moderate biofilm-forming abilities, and 20 were identified as weak producers. The isolates were classified based on their biofilm-forming capabilities, ranging from high to low (Table S9).

### Verification of sub-MIC non-growth-inhibitory activity

The influence of the selected sub-inhibitory concentration of the goldenseal fraction, corresponding to 1/2 MIC, on the planktonic viability of *P. aeruginosa* was evaluated. The untreated control exhibited a viable count of 8.20 ± 0.08 log ₁₁ CFU/mL, whereas exposure to 1/2 MIC of the GS fraction resulted in a count of 8.09 ± 0.11 log ₁₁ CFU/mL. The difference between the treated and untreated cultures was not statistically significant (*p* > 0.05). These findings demonstrate that the selected GS concentration did not exert a measurable inhibitory effect on bacterial growth under planktonic conditions, thereby confirming its subinhibitory nature. Consequently, the observed suppression of biofilm formation may be attributed to antibiofilm activity rather than a reduction in bacterial viability of the cells.

### Molecular characterizations

Analysis of the 16 S rRNA gene sequences of the tested isolates P1, P2, and P3 demonstrated high similarity to *Pseudomonas aeruginosa* strain ATCC 10,145, with 99.05% sequence identity. Among the examined isolates, P1 displayed the highest antibiotic resistance profile and the strongest biofilm-forming capacity; therefore, it was selected for detailed molecular confirmation. The amplified 16 S rRNA gene sequence was analyzed using the BLASTn tool against sequences available in the NCBI GenBank database to verify species identity. Molecular identification supported the phenotypic characterization of the isolate as *P. aeruginosa*. The confirmed partial 16 S rRNA gene sequence of isolate P1 was submitted to GenBank under the accession number PV640469.

### Antimicrobial activity of GS on *P. aeruginosa*

Clear and well-defined zones of inhibition were observed around the wells on Mueller–Hinton agar plates inoculated with a 0.5 McFarland-standardized suspension of *Pseudomonas aeruginosa*. The diameters of these zones ranged from 19 to 24 mm after incubation at 37 °C for 24 h. The consistency and size of the inhibition zones indicated a pronounced antibacterial effect of the tested fraction against the isolate. These findings confirm the susceptibility of *P. aeruginosa* to the applied treatment and highlight the notable antimicrobial activity exhibited under the tested conditions, as shown in (Fig. S2, Supplementary File).

### MIC determination

The MIC of the goldenseal fraction was assessed against three selected multidrug-resistant *Pseudomonas aeruginosa* isolates, P1, P2, and P3, which demonstrated strong biofilm production. These isolates were coded as trials 1, 2, and 3 during the assay. A uniform MIC value of 3.125 mg/mL was recorded for all three isolates, corresponding to the minimum concentration of the fraction that inhibited the visible bacterial growth.

### Sub-Inhibitory concentration of GS on production of biofilm

The goldenseal fraction sub-MIC presented a high level of inhibition of biofilm production in an MDR strong-biofilm-producing *P. aeruginosa*. The three selected isolates (P1, P2, P3) showed decreases in biofilm production of 85%, 80%, and 60% in trials 1, 2, and 3, respectively, compared to the control. A significant degree of biofilm formation inhibition was observed.

### Influence of GS on lasR, lasI, Rh1I, and rhlR genes expression levels

**Fig. 5:**
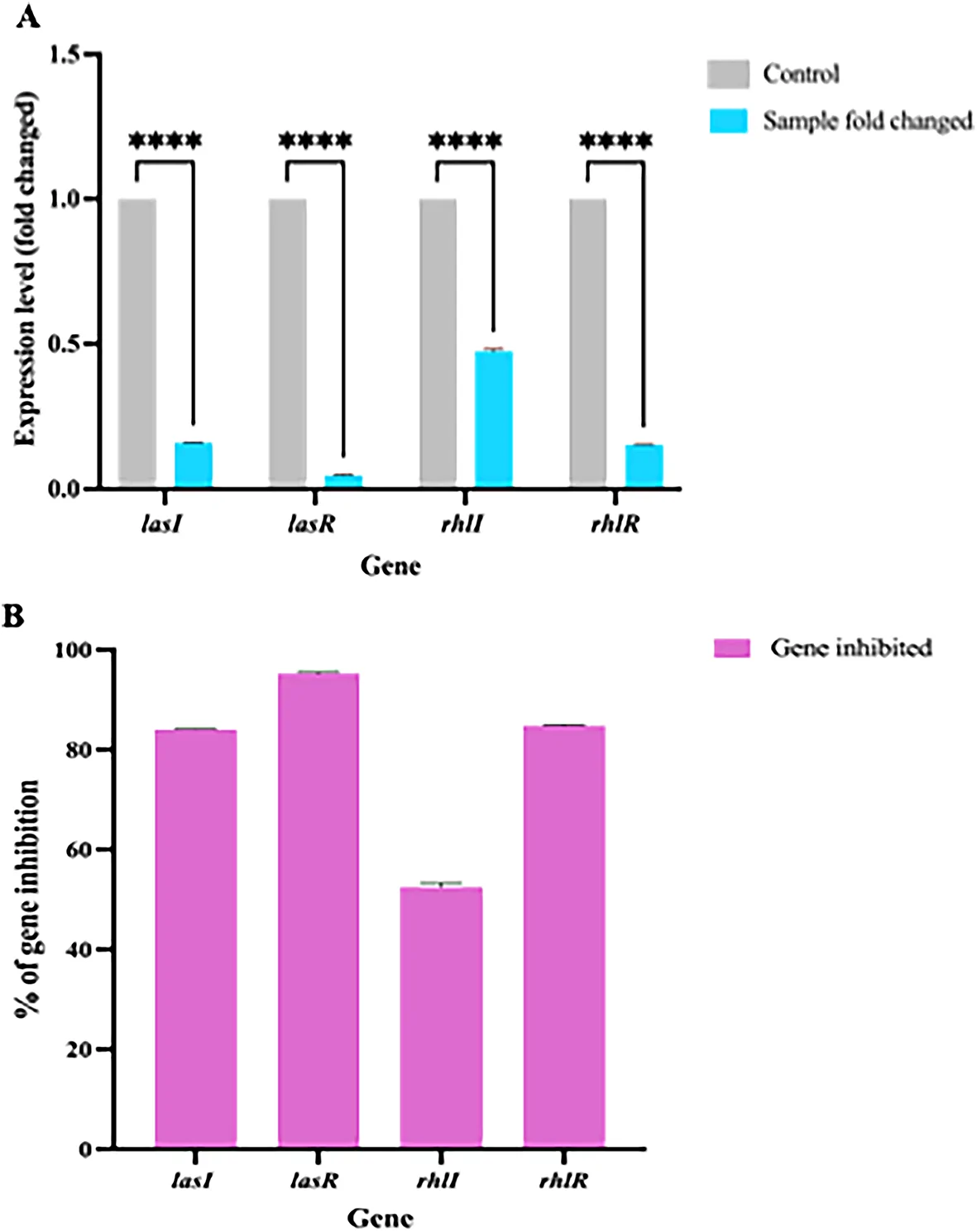
Effect of GS fraction on the transcriptional levels of quorum sensing genes (LasR, LasI, RhlR, and RhlI) in *P. aeruginosa* isolates. (**A**) Relative expression levels of quorum sensing genes in untreated control (gray) and GS-treated groups (cyan). Data are presented as mean ± SD. (**B**) Percentage inhibition of GS fraction on quorum sensing regulatory genes. LasR showed the highest inhibition, followed by RhlR and LasI, while RhlI showed the lowest inhibition. Statistical significance is defined as: *p < 0.05, **p < 0.01, ***p < 0.001, ****p < 0.0001 vs. control. The relative expression was calculated using the 2^-ΔΔCt method, where the untreated control was set to 1.0 (100%). The treated group exhibited a relative expression value of approximately 0.047, corresponding to 95.3% downregulation (i.e., 1–0.0461 = 0.953, or 95.3%).

Treatment with the goldenseal fraction markedly suppressed quorum-sensing gene expression in the three selected *P. aeruginosa* isolates (P1, P2, and P3). Among the investigated genes, *lasR* was the most strongly inhibited, showing 95.3% downregulation, followed by *rhlR* and *lasI*, which were reduced by 84.8% and 84.0%, respectively. The lowest inhibitory effect was observed for *rhlI*, with a 52.5% downregulation (Fig. 5A). Expression levels differed significantly between untreated controls and treated isolates (*p* < 0.0001), with all values normalized to *16 S rDNA* (Fig. 5B). Results are presented as mean ± standard deviation (SD). Statistical analysis was carried out using one-way analysis of variance (ANOVA) followed by Tukey’s HSD post hoc multiple-comparison test, with statistical significance defined at *p* < 0.05. (Figs. S3–S5, Supplementary Materials). Relative expression values were obtained using the 2^−ΔΔCt approach, with the untreated control group assigned a baseline value of 1.0. For instance, treatment reduced the relative expression to approximately 0.047, reflecting a 95.3% downregulation compared to the untreated control (Table S10, Supplementary File).

### In vivo effectiveness of topical and oral GS fraction administration in Infected second-degree burn wound healing

The progression of wound healing in rats with infected burn wounds treated with GS, both orally and topically, was monitored over 12 days and compared to that of control rats. The macroscopic wound appearance was documented on days 0, 2, 4, 8, and 12. On day 0, all groups (Burn, GS Gel, and GS Oral) exhibited no variation in wound sizes and appearance following burn induction. By day 2, all groups displayed intense inflammation with deep erythema and exudate, indicating ongoing infection. On day 4, the GS-treated groups exhibited a marked reduction in wound size and signs of epithelialization. The gel group showed more advanced tissue regeneration, with reduced necrotic tissue. The oral group also demonstrated visible progress, though less pronounced than the gel group. By day 8, wounds in the GS gel group showed significant closure and re-epithelialization, with only small, unhealed areas remaining. The Oral group also demonstrated marked improvement. In contrast, the Burn group retained large, inflamed wound beds with minimal closure and evidence of ongoing infection. On day 12, a complete or near-complete wound closure was noticed in the GS gel-treated rats, and restored skin integrity. The oral group also achieved notable healing, with minor residual scarring. Meanwhile, the burn group still exhibited incomplete healing, characterized by erythema, inflammation, and unhealed tissue. Overall, both topical (gel) and oral GS treatments significantly enhanced the quality and rate of wound healing in infected burn wounds, with topical GS application proving to be the most effective in reducing inflammation, promoting tissue regeneration, and achieving faster wound closure.

**Fig. 6:**
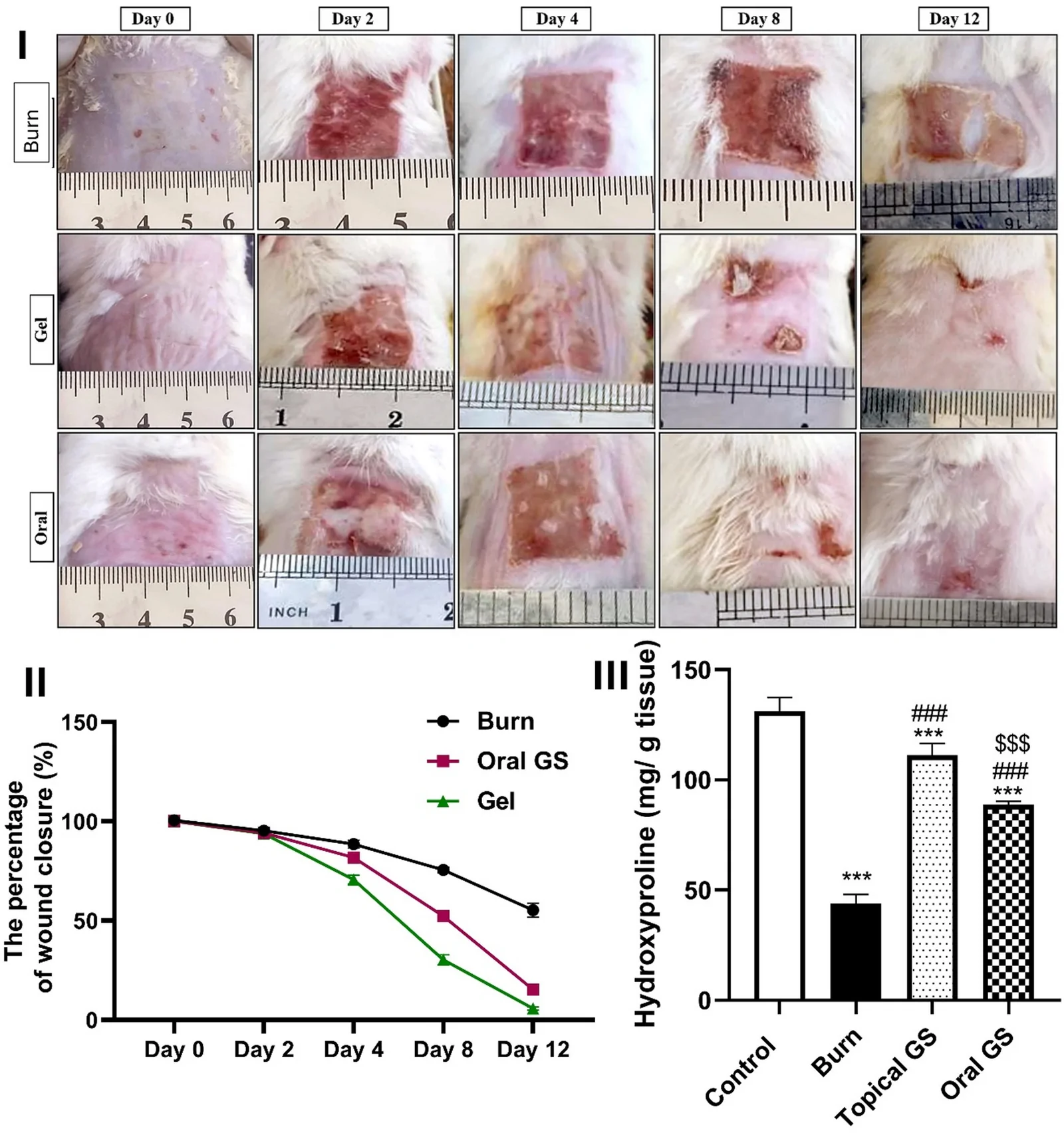
I- Macroscopic evaluation of wound healing progression in rats with infected burn wounds treated with GS orally and topically. Representative images of wound areas taken on Days 0, 2, 4, 8, and 12 post-injuries for three groups: Control (no treatment), Gel (topical GS treatment), and Oral (oral GS administration). At Day 0, similar burn wounds were induced in all groups. Rulers placed adjacent to the wounds were used to estimate wound size throughout the study period. II- Quantitative analysis of wound closure percentage over time in the three groups. Data are expressed as mean ± SV. III- Hydroxyproline content (mg/g tissue) in the skin of different groups, reflecting collagen deposition. Values are expressed as mean ± SD. ***P < 0.001 versus the control, ###*P* < 0.0001 versus the burn group, and ^$$$^*P* < 0.0001 versus the topical *goldenseal* group.

Quantitative analysis of wound closure over time revealed a substantial reduction in the wound area in all groups (Fig. 6I–II). However, both topical gel and oral GS treatments markedly increased the rate of wound closure compared to the untreated burn group. Notably, by day 12, the topical GS group exhibited the highest percentage of wound closure, indicating superior healing efficacy (*p* < 0.0001). Furthermore, as an indicator of collagen synthesis and tissue remodeling, hydroxyproline levels were measured in the skin tissue samples (Fig. 6III). The burn group exhibited a marked decline in hydroxyproline content in contrast to the healthy control rats (*p* < 0.001), reflecting impaired collagen deposition. Treatment with either topical gel or oral GS significantly restored hydroxyproline levels (*p* < 0.001 vs. burn). Moreover, the group treated with topical GS demonstrated a significantly elevated hydroxyproline content compared to the group administered oral GS (*p* < 0.001), indicating improved collagen regeneration with topical GS application.

### Histopathological evaluation of topical and oral administration of GS fraction against infected burn wound healing

**Fig. 7:**
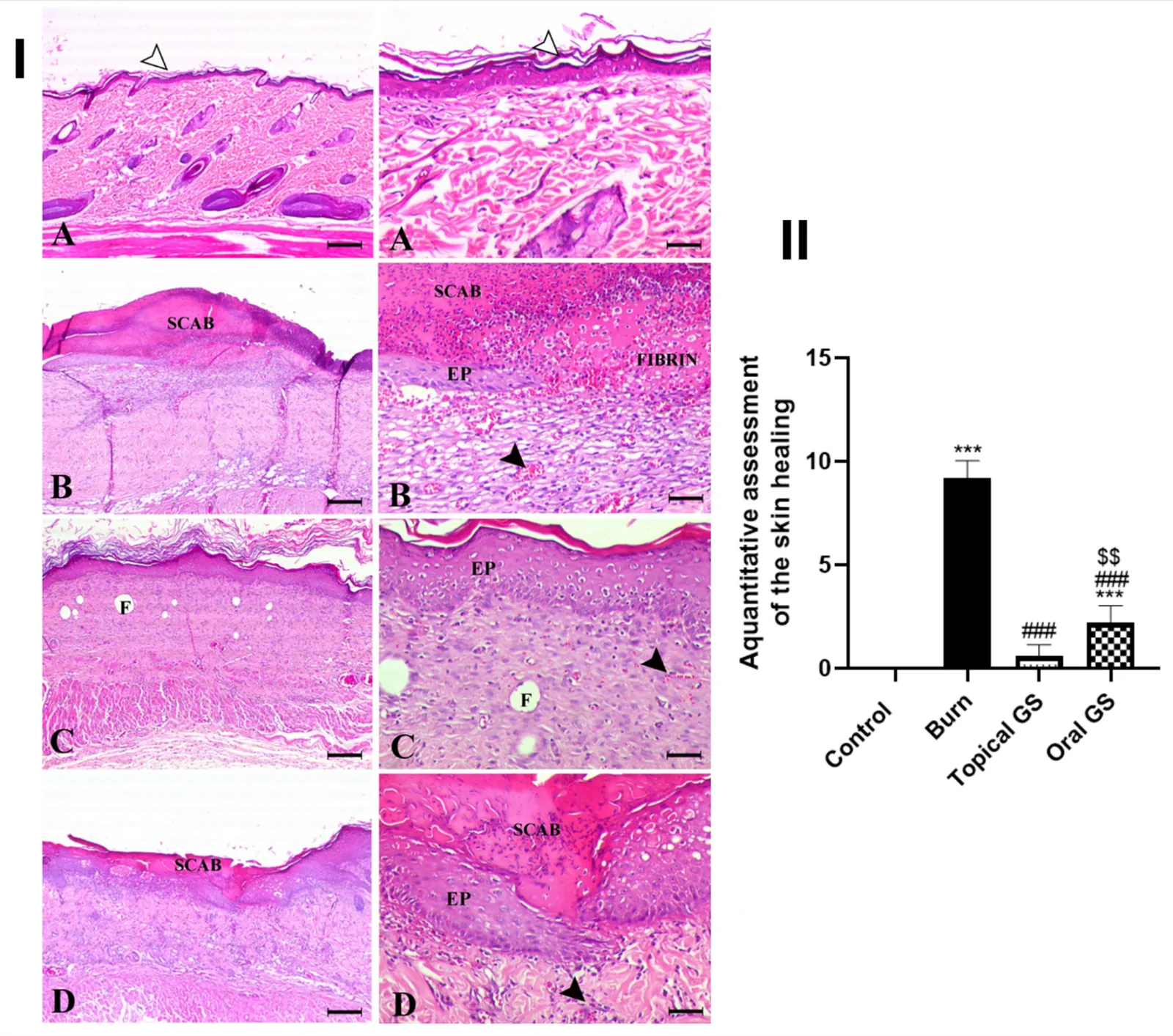
I- Representative photomicrographs of skin sections from different animal groups: (**A**) Control group showing normal skin tissues with intact epidermis and dermis (white arrowheads indicate normal epidermal covering). (**B**) Burn-infected group displaying features of delayed wound healing, characterized by a thick scab, marked fibrin deposition, and extensive angiogenesis (black arrowheads). (**C**) Burn-infected group treated with topical application of *goldenseal* fraction, showing marked re-epithelialization and dermal remodeling (F indicates fat globules within the dermis). (**D**) Burn-infected group treated with oral administration of GS, showing incomplete epithelial coverage with residual scab and reduced angiogenesis (black arrowheads). H&E stain; scale bars = 200 μm (left column) and 50 μm (right column). II-A quantitative assessment of skin healing in different groups. Values are expressed as mean ± SD. ***P < 0.001 versus the control, ^###^*P* < 0.0001 versus the burn group, and ^$$^*P* < 0.001 versus the topical *goldenseal* group.

The skin of normal control animals exhibited normal histological architecture of both the epidermis and dermis. The dermis contained various connective tissue elements, including collagen and elastic fibers, and included normal epithelial components such as hair follicles, sweat glands, and other skin appendages. The epidermis comprised stratified squamous keratinized epithelium, serving as the protective outer covering (Fig. 7I–A). In the infected, non-treated group, the skin still showed a protective scab, characterized by a thick, eosinophilic, structureless layer underlain by a fibrin-rich matrix. There was marked angiogenesis and dense leukocytic infiltration, predominantly composed of neutrophils, lymphocytes, and macrophages. Most animals exhibited a limited epithelial tongue at the wound margin, with a wide central gap devoid of epithelial covering, indicative of impaired re-epithelialization (Fig. 7I–B). Burn-injured animals treated with topical application of goldenseal fraction showed complete re-epithelialization with full-thickness epithelial coverage. The dermis exhibited marked remodeling of collagen fibers, minimal angiogenesis, and a complete absence of inflammatory infiltrates. Some areas displayed absorbed fat globules within the dermis without any associated inflammatory reaction (Fig. 7I–C). In contrast, burn-injured animals treated with oral administration of *the* goldenseal fraction demonstrated a notable reduction in inflammatory and angiogenic features within the dermis, along with evidence of re-epithelialization. However, the epithelial coverage in most animals remained incomplete (Fig. 7I–D). Quantitative scoring of skin healing supported the histological observations. The burn group exhibited a marked elevation in injury score in contrast to the control (*p* < 0.001), indicating severe tissue damage. Both treatment groups (topical and oral GS) showed significantly reduced injury scores in contrast to the burn group (*p* < 0.001). Notably, topical GS treatment resulted in significantly better healing scores than oral GS treatment, suggesting the enhanced topical efficacy of GS in promoting skin regeneration (Fig. 7II). The increased hydroxyproline content in GS topical treatment was greater than that in the oral route, consistent with enhanced collagen deposition. This was supported by histopathological findings (H&E), which showed improved collagen organization and dermal remodeling in the topical GS-treated wounds compared to the oral group.

### Modulation of inflammatory cytokines by GS treatment in infected burn-induced skin injury

**Fig. 8:**
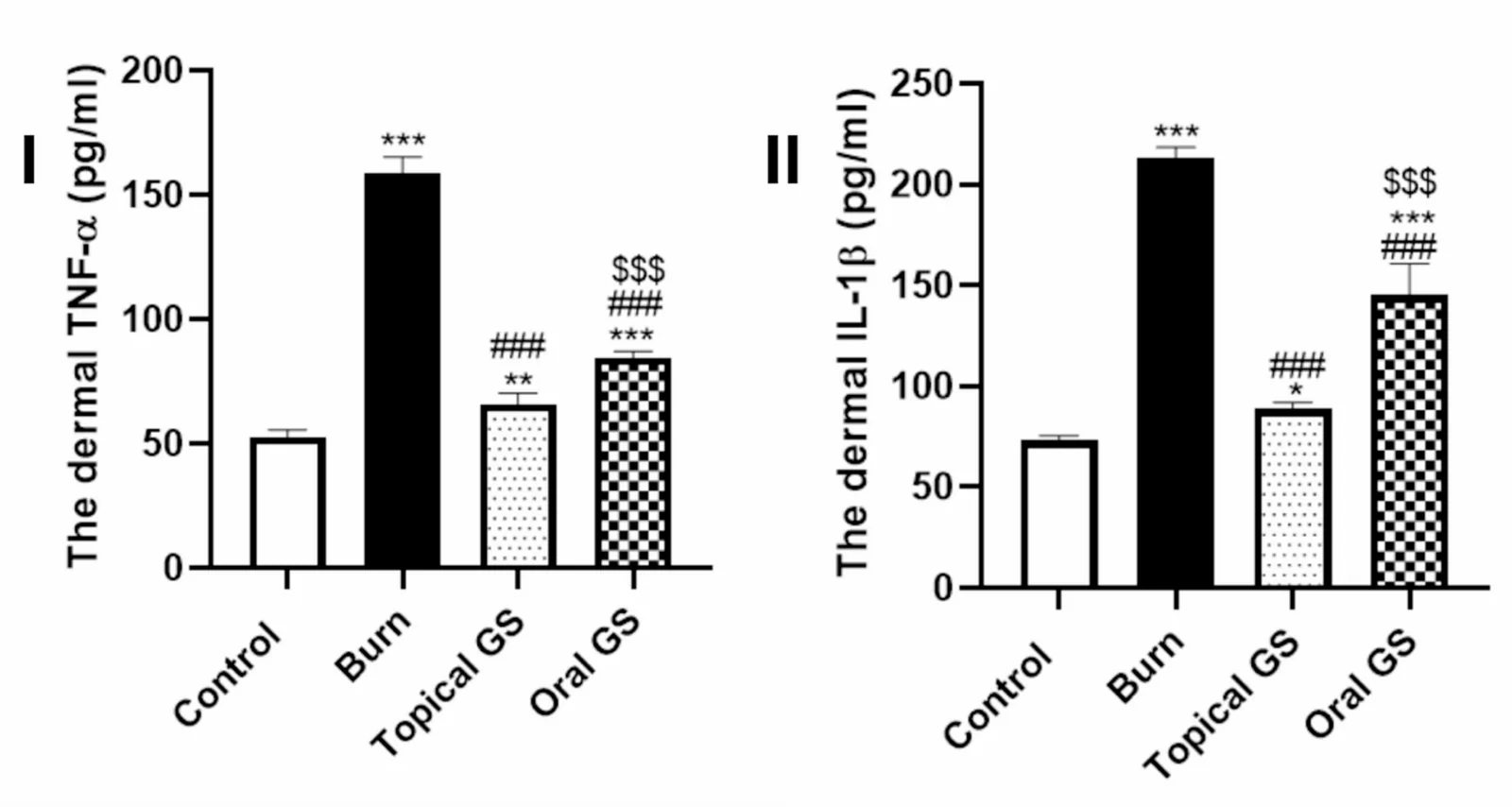
*Goldenseal* fraction (GS) treatment attenuates burn-induced dermal inflammation by reducing TNF-α and IL-1β levels. (I) Levels of tumor necrosis factor-alpha (TNF-α) and **(**II**)** interleukin-1 beta (IL-1β) were quantified in skin tissue homogenates from control, burn, topical GS-treated, and oral GS-treated groups using ELISA. Burn injury significantly increased both cytokines compared to the control. Topical and oral GS treatments significantly reduced cytokine levels, with topical GS showing a more significant effect. Data are expressed as mean ± SD (*n* = 6). ***P < 0.001, **P < 0.01, and *P < 0.05 versus control, ^###^*P* < 0.001 versus the burn group, and ^$$$^*P* < 0.001 versus the topical *golden seal* group.

To evaluate the anti-inflammatory response and possible antibacterial effects associated with GS treatment, dermal levels of the pro-inflammatory cytokines interleukin-1β (IL-1β) and tumor necrosis factor-α (TNF-α) were quantified in skin tissues from all experimental groups. As depicted in Fig. 8I, burn injury resulted in a significant elevation of TNF-α levels compared to healthy control rats (*p* < 0.001), indicating a marked inflammatory response. Both topical and oral GS treatments significantly decreased TNF-α concentrations compared to the untreated burn group (*p* < 0.001). Notably, topical GS administration led to a greater reduction, suggesting a more pronounced local anti-inflammatory effect than systemic GS administration. Similarly, as shown in Fig. 8II, IL-1β concentrations were noticeably increased in burn group compared to the control group (*p* < 0.001). Treatment with topical and oral GS significantly reduced IL-1β expression (*p* < 0.0001), with a more prominent reduction shown by the topical application. These findings indicate that both topical and oral GS exert anti-inflammatory effects in burn-injured skin, with topical administration demonstrating more significant efficacy. The substantial reduction in TNF-α and IL-1β levels suggests that GS may possess antibacterial potential, as these cytokines are key mediators of inflammation in response to bacterial infection (Fig. 8).

### The antibacterial effects of topical and oral GS on burn-induced changes in CAMP and RBD2

**Fig. 9:**
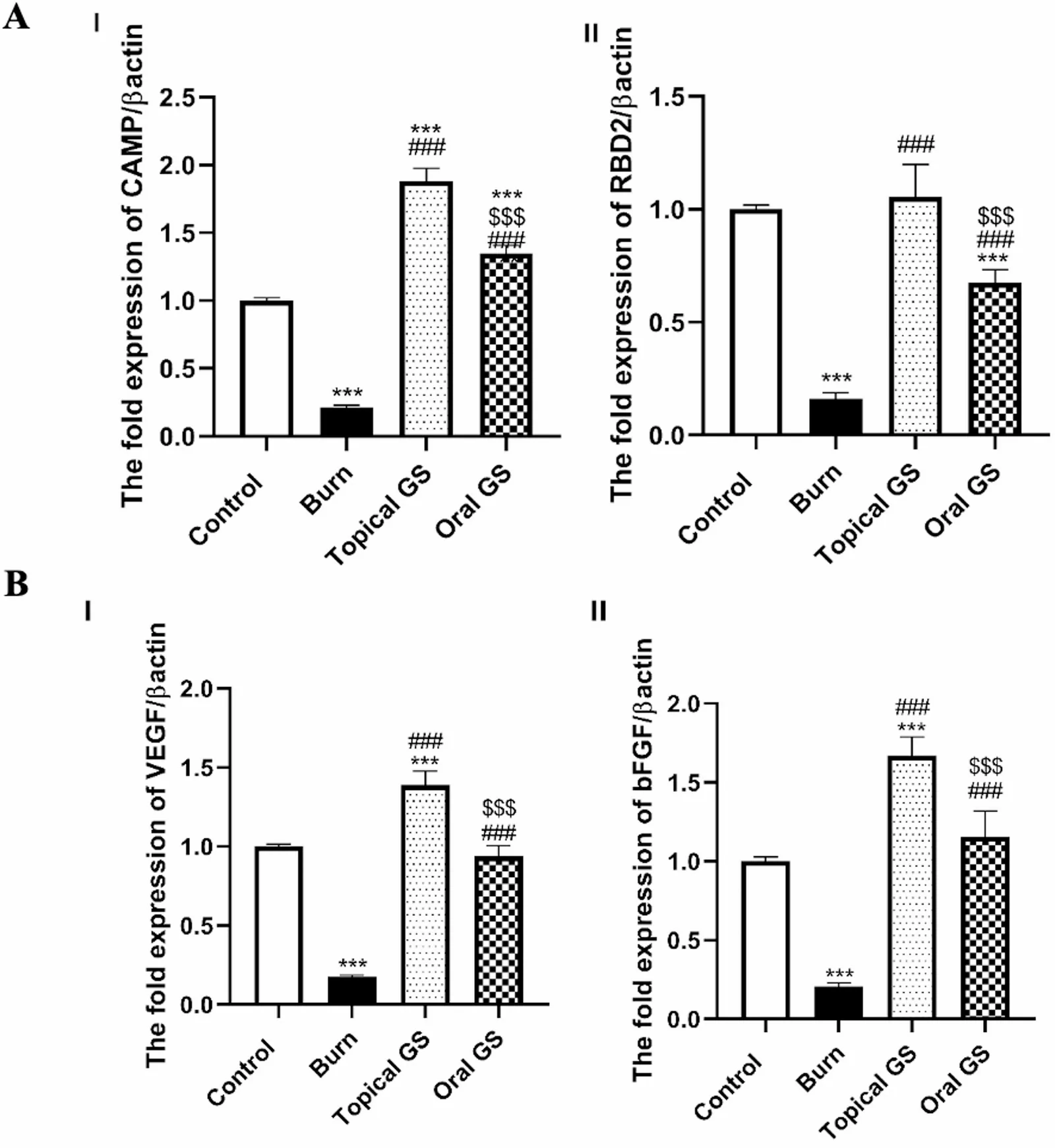
**A**: Effects of burn injury and GS treatment on the relative mRNA expression of cathelicidin antimicrobial peptide (CAMP) (I) and rat β-defensin 2 (RBD2) (II) in skin tissues. **B**: Effects of GS treatment on the relative mRNA expression of vascular endothelial growth factor (VEGF) (I) and beta fibroblast growth factor (bFGF) (II) in skin tissues. Data are expressed as fold change relative to β-actin and presented as mean ± SD. ***p < 0.001 vs. control; ###*p* < 0.001 vs. Burn; $$$*p* < 0.001 vs. topical GS.

At mucosal surfaces, host defense partly relies on antimicrobial peptides produced by epithelial cells. Belonging to a distinct family of antimicrobial peptides, cathelicidins (CAMP) are distinguished by conserved pro-peptide domains and have been discovered in various mammals. Another antibacterial peptide is rat β-defensin 2 (RBD2), which displays an extensive antimicrobial role, particularly targeting gram-negative bacteria and molds, and is chiefly localized in mucosal epithelial cells of the bronchus, skin, and urogenital tract. To evaluate the antibacterial role of topical and oral GS administration in infected burn rats, their effect on the gene expression of antibacterial peptides CAMP and RBD2 was assessed using RT-PCR (Fig. 9A).

Our results revealed that burn injury exhibited a marked downregulation of CAMP mRNA expression, in contrast to the healthy rats (*p* < 0.001). Treatment with topical GS markedly enhanced CAMP expression, showing a marked increase in contrast to the burn group (*p* < 0.0001), with levels exceeding those of the control (*p* < 0.001), suggesting its significant antibacterial role. Oral GS treatment also significantly restored CAMP expression compared to the burn group (*p* < 0.001), although its effect was less than that of the topical GS group (*p* < 0.001). In the same line, Fig. 5II illustrated that burn injury caused a significant downregulation of RBD2 expression, in contrast to the healthy rats (*p* < 0.001). Administration of Topical GS significantly restored RBD2 expression to near-control levels (*p* < 0.001 vs. Burn), whereas Oral GS also increased RBD2 expression but to a lesser extent (*p* < 0.001). Together, these findings indicate that burn injury dysregulated CAMP and RBD2 expression, while GS treatment alleviates these alterations, with oral GS exerting stronger effects on CAMP normalization and topical GS showing superior recovery of RBD2 expression.

### The effects of GS treatment on the expression of angiogenic markers VEGF and bFGF in infected wound rats

To further assess the effect of GS administration in improving wound healing, we measured the mRNA expression of vascular endothelial growth factor (VEGF) and beta fibroblast growth factor (bFGF) using the RT-PCR technique. For VEGF expression **(**Fig. 9B–I**)**, burn injury caused a marked suppression in its mRNA expression compared to healthy rats (*p* < 0.001). Topical GS treatment markedly increased VEGF gene expression in contrast to the burn group (*p* < 0.001), reaching levels higher than the control (*p* < 0.001). Oral GS also significantly upregulated VEGF in contrast to the burn group (*p* < 0.001), though its effect was less pronounced than topical GS (*p* < 0.001). For bFGF expression (Fig. 9B–II), a similar pattern was observed. Burn injuries were associated with a significant decrease in bFGF expression compared to that in the control group (*p* < 0.001). Topical GS significantly increased bFGF expression beyond the levels observed in the burn group (*p* < 0.001), even surpassing those in the control group. Oral GS also notably increased bFGF levels compared to the burn group (*p* < 0.001), although the expression was still considerably lower than that achieved with topical GS (*p* < 0.001). Our findings suggest that GS treatment, especially in its topical form, effectively counteracts burn-induced suppression of angiogenic factors VEGF and bFGF, thereby enhancing angiogenesis and tissue repair.

**Fig. 10:**
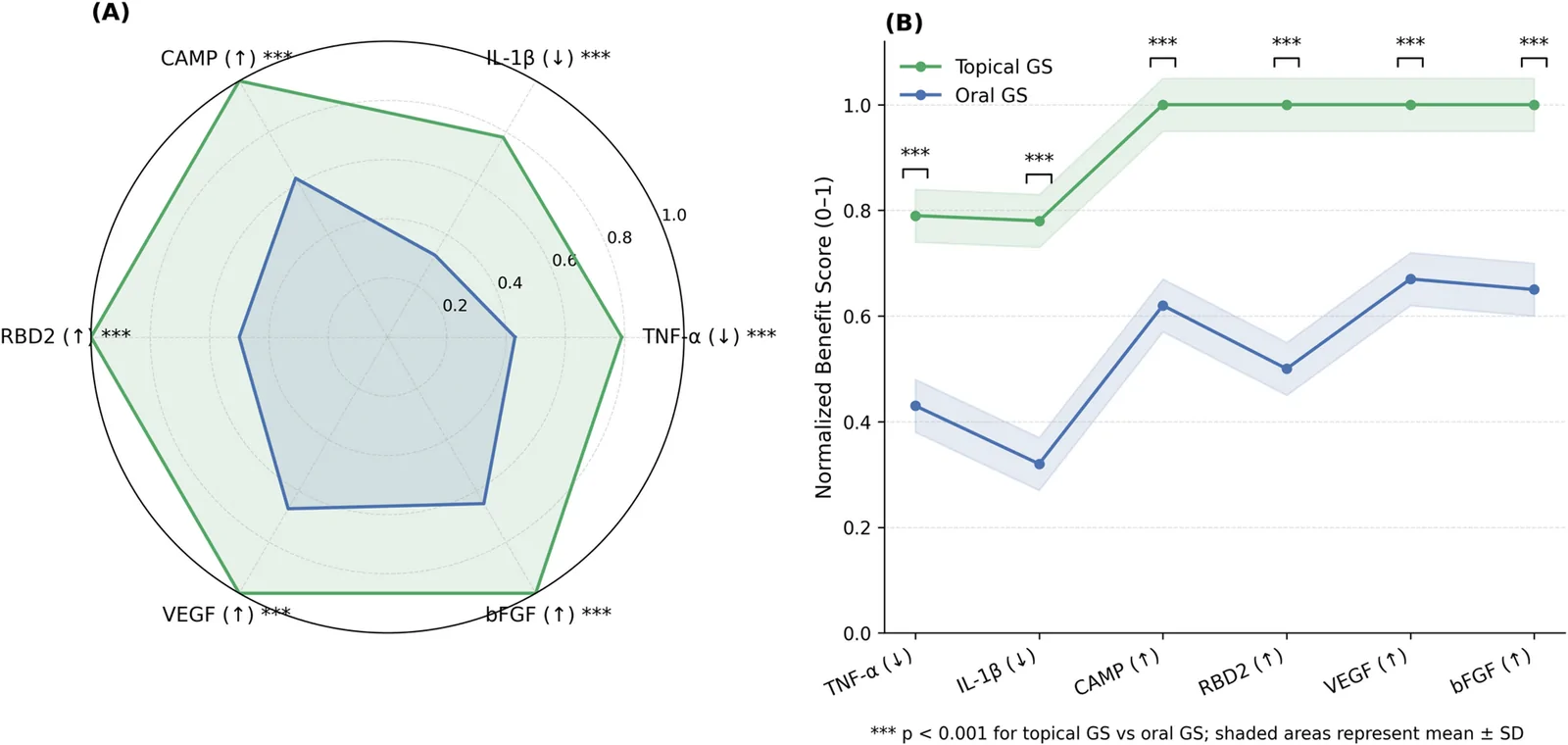
Comparative profiling of oral and topical Goldenseal across inflammatory and regenerative markers. (A) Radar plot showing normalized benefit scores (0–1; higher values indicate stronger effects) for TNF-α, IL-1β, CAMP, RBD2, VEGF, and bFGF. Statistical significance between topical and oral treatments is indicated for each parameter (***p < 0.001). (B) Line plot showing treatment responses across the same parameters. Pairwise comparisons between topical and oral Goldenseal are indicated above each marker (***p < 0.001). Shaded areas represent mean ± SD.

Comparative profiling highlighted the clear advantage of topical goldenseal over oral administration in terms of inflammatory and regenerative parameters. Radar analysis demonstrated uniformly higher benefit scores for topical treatment, with the most pronounced effects on cytokine suppression (TNF-α and IL-1β) and angiogenic markers (VEGF and bFGF) (Table S11, Supplementary file). Consistently, profile plots with confidence-style bands confirmed that topical goldenseal maintained superior outcomes across all measured axes, underscoring its stronger anti-inflammatory and pro-healing potential compared with oral delivery (Fig. 10).

## Discussion

Burn injuries represent one of the most frequent and severe forms of trauma, resulting from physical or chemical insults and contributing significantly to global mortality^36^. The increasing prevalence of multidrug-resistant *P. aeruginosa* in healthcare-associated infections, especially in wounds and burns, underscores the urgent need for alternative and effective antimicrobial strategies to combat these infections. *P. aeruginosa* is particularly problematic because of its intrinsic resistance mechanisms, ability to acquire additional resistance determinants, and capacity to form robust biofilms that protect it from host defenses and antimicrobial agents. These characteristics not only complicate treatment but also prolong hospital stays and increase the risk of systemic infection. Consequently, there is growing interest in exploring novel therapeutic approaches, including plant-derived compounds and natural products, which may offer safer and more effective alternatives that target both bacterial growth and virulence factors^37^.

The phytochemical composition of the active goldenseal fraction was characterized using liquid chromatography coupled with electrospray ionization tandem mass spectrometry (LC–ESI–MS/MS). A total of 52 bioactive metabolites were identified, including phenolics, flavonoids, alkaloids, nucleosides, and organic acids. Compound identification was confirmed by comparing retention times, precursor ions (Q1), and characteristic product ion spectra (Q3) with authentic standards and literature references. This comprehensive chemical fingerprint provided the basis for selecting 52 representative compounds for molecular docking studies and functional validation.

Molecular docking revealed the quorum-sensing (QS) inhibitory potential of the goldenseal-derived phytochemicals. Naringenin-7-O-glucoside exhibited the strongest interaction with the *LasR* receptor, suggesting its potential as a lead scaffold for QS inhibition. Other ligands, including hydrastinine, cis-trans-abscisic acid, inosine, and berberine, also exhibited relevant binding affinities. These findings align with prior reports on flavonoid and alkaloid derivatives as QS modulators, underscoring the role of plant secondary metabolites in disrupting bacterial communication^38–40^.

Orotic acid exhibited the strongest binding to the *LasI* receptor, followed by galacturonic, chlorogenic, gallic, and oxypurinol. *LasI* showed weaker binding affinities than the other receptors, supporting the view that *LasR* occupies a more central and responsive part in communication via the QS hierarchy of *P. aeruginosa*^41^. The RhlR receptor showed strong interactions with naringenin-7-O-glucoside and daidzein, followed by chlorogenic acid, mannose-6-phosphate, hydrastinine, and allocryptopine, indicating the potential for synergistic inhibition by flavonoids and phenolic acids^42^. In contrast, RhlI displayed the highest affinity for chlorogenic acid, followed by orotic acid, daidzein, naringenin-7-O-glucoside, and guanine. Although the overall affinities were lower than those observed for *LasR* and RhlR, the recurrent activity of chlorogenic acid across multiple receptors highlights its potential as a versatile quorum-sensing inhibitor. Taken together, these results identify *LasR* as the most responsive receptor, with naringenin-7-O-glucoside exhibiting the strongest overall binding affinity. In contrast, *LasI* exhibited the weakest interactions, with only orotic acid displaying a notable affinity. This highlights the central role of *LasR* in QS and biofilm regulation in *P. aeruginosa* and underscores the potential of goldenseal-derived phytochemicals as anti-biofilm therapeutic agents.

These computational predictions were supported by in vitro findings, where the goldenseal fraction significantly inhibited biofilm formation and downregulated virulence gene expression. This fraction interfered with bacterial adhesion, disrupted biofilm structure, and modulated the microenvironment, thereby impairing biofilm-associated pathways. Although direct studies on *P. aeruginosa* biofilms remain limited, comparable inhibitory effects on other bacterial species further support the potential of goldenseal as an antibiofilm agent^3^. All 100 clinical *P. aeruginosa* isolates exhibited complete resistance to ticarcillin and piperacillin, with additional resistance to ceftazidime, amikacin, and cefepime, indicating broad multidrug resistance. Biofilm formation at wound and burn sites further complicates treatment, underscoring the urgent need for novel therapeutic strategies capable of disrupting biofilms and enhancing wound healing^8^. To our knowledge, no study has directly linked goldenseal with any specific antibiofilm formation or downregulation of virulence genes responsible for biofilm formation in *P. aeruginosa*. Sub-MIC treatment with the GS fraction significantly reduced biofilm formation and downregulated key quorum-sensing genes in *P. aeruginosa*, with the greatest effects on *lasR*, *rhlR*, and *lasI* expression. These findings support the hypothesis that goldenseal disrupts quorum sensing, thereby limiting biofilm maturation and virulence. Importantly, this antivirulence mechanism reduces pathogenicity without imposing the selective pressure associated with conventional antibiotics. Thus, the availability of a low-cost, convenient, and less toxic plant-derived antimicrobial agent is highly valuable. It has a significant effect on virulence factors in critical multidrug-resistant bacteria, such as *Pseudomonas aeruginosa*. This may contribute to reducing the morbidity and mortality associated with infections caused by these pathogens. Goldenseal likely interferes with bacterial communication systems, rather than directly inhibiting growth. This distinction is important because targeting quorum sensing can weaken coordinated activities, such as biofilm formation and the expression of virulence factors, without exerting strong evolutionary pressure that typically drives antibiotic resistance. In the case of *Pseudomonas aeruginosa*, a pathogen known for its adaptability and resistance, such an approach could limit its ability to establish persistent infections. Despite these promising observations, additional studies are needed to clarify the underlying molecular interactions, determine the consistency of the effects across different strains, and evaluate their performance under physiological conditions.

The disruption of quorum sensing by the GS fraction was further validated in an in vivo burn model, where both oral and topical administration accelerated wound contraction and restored hydroxyproline levels. Topical treatment demonstrated superior effects on epithelialization and collagen remodeling, indicating that GS not only exerts antimicrobial activity but also promotes tissue repair and regeneration in the skin.

In clinical practice, the primary objective of burn management is to accelerate epithelialization and skin regeneration while preventing wound infection^43^. This complex process progresses through three overlapping phases: inflammation, cellular proliferation, and tissue maturation^44,45^. The percentage of wound closure is a critical indicator of burn healing progression^46^. In this study, macroscopic evaluation and quantitative wound size measurements demonstrated a significantly accelerated closure in the GS group compared with untreated burns. Topical application was particularly effective, yielding faster wound contraction and higher collagen content, as indicated by increased hydroxyproline levels. Collagen synthesis is essential for wound tensile strength and remodeling^47^, and hydroxyproline, a key collagen constituent, is widely used as a biomarker for matrix deposition during tissue repair^32^. Both oral and topical GS administration increased hydroxyproline production compared to the control group, indicating enhanced tissue repair. The most pronounced effects were observed in the topical application group. Histological analysis revealed complete re-epithelialization and dermal remodeling following topical application, whereas oral treatment resulted in only partial recovery accompanied by moderate inflammation. The superior efficacy of topical GS is likely due to the higher local concentrations of bioactive constituents at the wound site, promoting regeneration and exerting localized antimicrobial activity.

Immediately following a burn injury, the affected arteries undergo transient vasoconstriction, which is rapidly followed by vasodilation to increase blood flow to the damaged tissue and initiate an inflammatory phase. This phase is characterized by the infiltration of immune cells, including neutrophils, mast cells, and basophils, as well as the release of inflammatory mediators such as prostaglandins, cytokines, and interleukins. In the presence of bacterial infection, excessive recruitment of neutrophils can exacerbate tissue damage and prolong inflammation, thereby impairing wound repair. This dysregulated response often manifests as intense pain during the early stages of healing and contributes to excessive scar tissue formation at the wound site^48^.

Analysis of inflammatory cytokines revealed marked elevations of TNF-α and IL-1β in burn wounds, consistent with the inflammatory cascade triggered by injury and bacterial infections. Both oral and topical GS treatments significantly reduced the levels of these pro-inflammatory mediators, with the latter producing the strongest effect. This reduction is clinically relevant because excessive inflammation delays repair and exacerbates tissue damage^49^. The immunomodulatory effects of GS, likely facilitated by alkaloids such as berberine, dampen inflammatory signaling and curtail secondary injuries^50^. Previous studies have established the anti-inflammatory efficacy of GS against P. gingivalis^51^.

An additional noteworthy discovery involved the alteration of antimicrobial peptides, specifically cathelicidin antimicrobial peptide (CAMP) and rat β-defensin 2 (RBD2), which are integral to the innate immune system’s defense mechanisms against pathogens^52^. Burn injuries markedly suppress their expression, thereby compromising the skin antimicrobial barrier^53^. GS treatment restored these peptides, with topical application demonstrating superior efficacy compared to oral administration. The upregulation of CAMP and RBD2 suggests a synergistic antimicrobial effect that may lower the microbial burden and facilitate wound repair. The antimicrobial activity of goldenseal has traditionally been attributed to its isoquinoline alkaloids, hydrastine, berberine, and canadine^10,54,55^. The elevated CAMP expression observed in the topical GS group beyond control levels likely represents a high stimulatory effect on host defense mechanisms, as goldenseal is rich in bioactive phytochemicals, including alkaloids and phenolic compounds, which are known to modulate innate immunity and promote the upregulation of antimicrobial peptides as part of the protective response^56^. This enhanced expression suggests that GS may not only restore compromised antimicrobial defenses following burn injury but also augment baseline innate immunity, thereby supporting more effective microbial clearance and improved wound healing. These findings align with the recognized immunomodulatory and pro-reparative properties of plant-derived compounds^57^. However, evidence indicates that crude fractions exhibit stronger activity than isolated berberine, implying synergistic contributions from additional phytochemicals^14,48^. Our findings are consistent with those of previous studies supporting the antimicrobial efficacy of GS^14,58^. Importantly, in addition to restoring antimicrobial defense, GS promotes pro-healing pathways, including the upregulation of angiogenic factors, further reinforcing its therapeutic value in burn wound management.

Angiogenesis, the formation of new blood vessels that supply oxygen and nutrients to regenerating tissues, is an essential component of tissue repair^59^. In the present study, GS bioactive fraction markedly increase the upregulation of the expression of angiogenic mediators VEGF and bFGF, both of which are critical for neovascularization and granulation tissue formation^60^. Burn wounds exhibited suppressed VEGF and bFGF levels, consistent with impaired healing, whereas GS administration, particularly via the topical route, restored their expression, thereby promoting angiogenesis and accelerating tissue repair. These findings are consistent with previous studies demonstrating that berberine, a key GS alkaloid, enhances angiogenesis through the activation of the HIF-1α/VEGF pathway, which improves outcomes in a rat model of cerebral ischemia-reperfusion injury^61^. These results indicate that GS not only attenuates infection and inflammation but also actively supports vascular remodeling, contributing to effective wound healing.

This study provides mechanistic and experimental evidence supporting the use of goldenseal (GS) as a potential adjunctive therapy for multidrug-resistant *P. aeruginosa* burn infections. By integrating antimicrobial, anti-inflammatory, and pro-regenerative actions, GS demonstrated superior efficacy when administered topically, underscoring the importance of localized delivery in optimizing therapeutic outcomes. These findings highlight the potential of GS-derived fractions to transform burn wound care and address the global challenge of antimicrobial resistance. The therapeutic advantages are evidenced by significant structural improvements in wound architecture, along with significant decreases in pro-inflammatory cytokines (IL-1β and TNF-α), and a notable increase in host defense peptides (CAMP, RBD2) and angiogenic mediators (VEGF, bFGF). This indicates that the antimicrobial properties of GS are effectively augmented by its immunomodulatory and healing-promoting capabilities, which facilitate tissue repair in infected burn environments. The present study provides scientific evidence supporting the use of GS fraction, particularly as a topical formulation, for the treatment of MDR *P. aeruginosa* burn infections. By targeting multiple pathways, including quorum sensing inhibition, inflammation control, antimicrobial defense enhancement, and angiogenesis stimulation, GS demonstrates substantial potential as an adjunctive therapy in burn wound management. This study offers a distinct contribution by demonstrating the multi-targeted therapeutic profile of goldenseal, which extends beyond conventional antimicrobial activity. Unlike traditional approaches that primarily focus on bacterial eradication, the GS fraction simultaneously interferes with quorum sensing, modulates inflammatory responses, enhances innate immune defense, and promotes angiogenesis and tissue regeneration. The integration of these effects within a single plant-derived formulation, particularly with enhanced efficacy through topical application, highlights a novel and comprehensive strategy for managing multidrug-resistant *P. aeruginosa* burn infections. This combined anti-virulence and pro-healing approach underscores the innovative nature of our findings and supports the potential of GS as a next-generation adjunctive therapy for burn care.

In summary, while previous studies have reported selected alkaloids and phenolic constituents of *Hydrastis canadensis*^62–64^, this study provides a comprehensive LC–ESI–MS/MS-based metabolite profile encompassing 52 phytochemicals across multiple chemical classes, including the underreported flavonoids and glycosides. Importantly, this study extends beyond phytochemical characterization by integrating target-based molecular docking against quorum-sensing regulators, in vitro antibiofilm and gene expression analyses, and in vivo wound-healing validation. To the best of our knowledge, this is the first study to link the broad-spectrum metabolite profile of goldenseal with quorum-sensing inhibition and therapeutic efficacy against MDR *P. aeruginosa* burn infections within a unified experimental framework.

A balanced and rigorous interpretation of the present findings should consider several important factors. Although in silico docking analysis provides valuable preliminary insights into potential ligand–target interactions, this approach is inherently based on static protein conformations and simplified scoring models; therefore, it may not fully reflect molecular flexibility, ligand dynamics, or the complexity of physiological systems. In addition, while the study supports a quorum-sensing–associated anti-virulence effect, direct in vitro and biochemical confirmation of quorum-sensing inhibition, particularly through quantification of QS signaling molecules, was beyond the scope of the current work. Similarly, the quantitative determination of the bacterial burden within infected tissues would further strengthen the assessment of in vivo antibacterial efficacy. Furthermore, although previous reports have described the potential adverse effects of goldenseal at high exposure levels^65^, a dedicated evaluation of systemic toxicity under the specific experimental conditions used in this study has not been performed. Addressing these aspects in future investigations will further refine the mechanistic interpretation, safety profile, and translational relevance of the present findings.

## Conclusion

This study provides comprehensive evidence that the goldenseal (GS) fraction represents a valuable biotechnological resource with multifaceted therapeutic potential against multidrug-resistant *Pseudomonas aeruginosa* in a second-degree burn model. In silico docking identified strong phytochemical–target interactions, notably naringenin-7-O-glucoside with *LasR*, corroborated by in vitro suppression of biofilm formation and quorum-sensing gene expression. In vivo validation demonstrated that GS, particularly via topical delivery, enhanced wound contraction, collagen remodeling, and epithelialization while modulating inflammatory cytokines (TNF-α, IL-1β), host-defense peptides (CAMP, RBD2), and angiogenic factors (VEGF, bFGF). By integrating computational, molecular, and translational approaches, this study underscores the biotechnological potential of plant-derived metabolites as adjunctive therapies that combine quorum sensing inhibition with anti-inflammatory and pro-regenerative mechanisms for managing MDR burn infections.

These findings highlight the promising role of safe plant-derived antimicrobials as alternative or complementary strategies against multidrug-resistant pathogens. By targeting key virulence determinants in *Pseudomonas aeruginosa*, such agents may help attenuate bacterial pathogenicity while minimizing toxicity and cost compared with conventional treatments. This approach could support improved infection control and therapeutic outcomes, particularly in resource-limited settings such as ours. Further investigations, including in vivo validation and mechanistic studies, are warranted to confirm their clinical applicability and optimize their use in combating resistant strains.

Overall, this study highlights the potential of goldenseal as a natural antivirulence agent against *P. aeruginosa*. By attenuating pathogenic traits instead of targeting bacterial survival, it offers a complementary strategy to conventional antimicrobial therapies. Its affordability and low toxicity profile further support its relevance as a candidate for future therapeutic development.

This study provides an in-depth, multi-layered evaluation of the antibiofilm and therapeutic potential of goldenseal against clinical isolates of Pseudomonas aeruginosa associated with second-degree burn infections. By integrating computational molecular docking, in vitro antimicrobial and antibiofilm assays, and in vivo validation in a burn model, the findings offer converging evidence of its efficacy across different experimental platforms. This comprehensive approach strengthens the reliability of the observed outcomes and highlights the relevance of goldenseal as a promising candidate for managing difficult-to-treat *P. aeruginosa* infections. The combined oral and topical assessment further underscores the potential translational value of this approach in burn wound therapy, supporting its future development as a complementary strategy to address biofilm-associated antimicrobial resistance.

To further advance the present findings and strengthen their translational relevance, future investigations should adopt rigorous and integrated experimental frameworks. Priority should be given to the targeted in vitro validation of the top-ranked compounds, with particular emphasis on confirming quorum-sensing (QS) inhibition through the direct quantification of signaling molecules and QS-regulated virulence determinants. Complementary functional QS assays, including reporter gene–based systems, along with mechanistic in vivo studies, would further clarify their effects on virulence regulation and biofilm formation.

In parallel, a detailed evaluation of membrane permeability, metabolic stability, and broader pharmacokinetic characteristics is essential to define the behavior of the identified bioactive compounds more precisely. Subsequent in vivo studies should incorporate a quantitative assessment of the bacterial burden in infected tissues, alongside comprehensive pharmacokinetic and pharmacodynamic analyses, to establish the therapeutic efficacy more robustly. Advanced infection models, such as bioluminescent systems, may provide additional value by enabling the real-time monitoring of disease progression and treatment response. Equally important is the implementation of well-controlled toxicity studies in mammalian models to delineate safety profiles and establish clinically relevant therapeutic windows.

From a translational perspective, further research should focus on the development and optimization of GS-based delivery platforms, including nanocarriers, hydrogels, and bioengineered wound dressings, to enhance bioavailability and target-site delivery. Integration with synthetic biology approaches and scalable bioprocessing strategies may additionally facilitate the efficient production of active phytochemicals. Rigorous clinical investigations are needed to establish the efficacy, safety, and regulatory viability of the GS fraction for human application. In addition, clinical evaluation will provide further evidence supporting its antimicrobial and anti-virulence activities, thereby reinforcing its therapeutic relevance and translational potential of this compound.

## Supplementary Information

Below is the link to the electronic supplementary material.

Supplementary Material 1

## Data Availability

The nucleotide sequence generated during this study (16 S rRNA gene of Pseudomonas aeruginosa isolate P1) has been deposited in the GenBank repository (NCBI), an INSDC member database, under accession number PV640469.https://www.ncbi.nlm.nih.gov/nuccore/PV640469.1/All other data supporting the findings of this study are included within the article and its supplementary information files.

## Abbreviations

ATCC: American type culture collection
bFGF: Basic fibroblast growth factor
BLASTn: Basic local alignment search tool (nucleotide)
CAMP: Cathelicidin antimicrobial peptide
CCASU: Culture collection of ain shams university
CLSI: Clinical and laboratory standards institute
CMC: Carboxymethyl cellulose
H&E: Hematoxylin and eosin
HIF-1α: Hypoxia-inducible factor-1 alpha
HSD: Honestly significant difference
IL-1β: interleukin-1 beta
ImageJ: ImageJ software (NIH)
LB: Luria–bertani
LC–ESI–MS/MS: Liquid chromatography–electrospray ionization–tandem mass spectrometry
MDR: Multidrug-resistant
MHA: Mueller–hinton agar
MIC: Minimum inhibitory concentration
MRM: Multiple-reaction monitoring
MS: Mass spectrometry
MS1/MS2: First/second stage mass spectra
NCBI: National center for biotechnology information
NIH: National institutes of healthOD: Optical density
ODc: Cut-off optical density
PBS: Phosphate-buffered saline
PDB: Protein data bank
qRT-PCR: Quantitative real-time
QS: Quorum sensing
RBD-2: Rat β-defensin-2
RMSD: Root-mean-square deviation
RNA: Ribonucleic acid
RT-PC: Real-time polymerase chain reaction
SPSS: Statistical package for the social sciences
TNF-α: alpha tumor necrosis factor
VEGF: Vascular endothelial growth factor
WDCM: World data centre for microorganisms
