# Supplementary material for: Bioactive goldenseal (Hydrastis canadensis) fraction suppresses multidrug-resistant Pseudomonas aeruginosa in a rat burn wound model: oral and topical administration
Source: Sci Rep. 2026 Sep 2;16:27506. doi: 10.1038/s41598-026-60341-z (PMC13538558; doi:10.1038/s41598-026-60341-z)
Supplement: Supplementary file 1 [file 41598_2026_60341_MOESM1_ESM.docx]

**Supplementary File

Bioactive goldenseal (*Hydrastis canadensis*) fraction suppresses multidrug-resistant *Pseudomonas aeruginosa* in a rat burn wound model: oral and topical administration**

**Table S1**

List of primers used for amplification of *Pseudomonas* 16S rDNA and quorum sensing genes *(lasI, lasR, rhlI,* and *rhlR*)

| **Reference** | **Primer sequence (5'-3')** | **Gene** |
| --- | --- | --- |
| [1] | GACGGGTGAGTAATGCCTA | Pseudomonas  16S rDNA |
|  | CACTGGTGTTCCTTCCTATA |  |
| [2] | ATGATCGTACAAATTGGTCGGC | *lasI* |
|  | GTCATGAAACCGCCAGTCG |  |
| [3] | CTGTGGATGCTCAAGGACTAC | *lasR* |
|  | AACTGGTCTTGCCGATGG |  |
|  | GTAGCGGGTTTGCGGATG | *rhlI* |
|  | CGGCATCAGGTCTTCATCG |  |
|  | GCCAGCGTCTTGTTCGG | *rhlR* |
|  | CGGTCTGCCTGAGCCATC |  |

**Table S2**

Forward (F) and reverse primer (R) sequences

| **Gene** | **Forward Primer Sequence (5'-3')** | **Reverse Primer Sequence (5'-3')** | **Accession number** |
| --- | --- | --- | --- |
| *VEGF* | GATCATGCGGATCAAACCTCACC | CCTCCGGACCCAAAGTGCTC | NP_446105.1 |
| *bFGF* | CAACACTTACCGGTCACGGA | AAGCTCTACCACAGGGGACT | NM_019305 |
| *RBD-2* | TTAATTTGGTTTGTTTTGTGCAT | CATGCCTGACCAAAGGAGGCGTA | AF068861 |
| *CAMP* | GCAGTTCCAGAGGGACGTC | GTTCCTTGAAGGCACATTGC | NM_001100724.1 |
| *β-actin* | GCATCTTCTTGTGCAGTGCC | TACGGCCAAATCCGTTCACA | NM_031144.3 |

**Table S3**

MRM parameters and identification of phenolic compounds in aqueous GS by LC-ESI-MS/MS

| **ID** | **Conc. (ug/g)** | **Q1**  **(m/z)** | **Q3**  **(m/z)** | **RT (min)** | **CE**  **(V)** | **CXP**  **(V)** | **DP**  **(V)** |
| --- | --- | --- | --- | --- | --- | --- | --- |
| Gallic acid | 4.72 | 168.9 | 124.9 | 1.69 | -30 | -11 | -110 |
| Coumaric acid | 1.89 | 162.9 | 119 | 7.7 | -20 | -7 | -90 |
| 3.4-Dihydroxybenzoic acid | 14.44 | 152.9 | 109 | 3.13 | -40 | -5 | -75 |
| Ferulic acid | 33.18 | 192.8 | 133.9 | 8.89 | -16 | -5 | -25 |
| Syringic acid | 11.03 | 196.9 | 181.9 | 6.23 | -12 | -5 | -30 |
| Chlorogenic acid | 3.78 | 353 | 191 | 5.1 | -23 | -10 | -60 |

Collision energy (CE). Collision cells exit potential (CXP) declustering potential (DP

**Table** **S4** LC–ESI-MS/MS scanning for identification of phenolic compounds in GS fraction using negative ion mode

| **ID** | **Metabolite name** | **Chemical Formula** | **RT (min)** | **m/z** | **M.wt** | **Fragments** | **S/N** | **Chemical Class** |
| --- | --- | --- | --- | --- | --- | --- | --- | --- |
| 1 | HydroxyButyric acid | C_4_H_8_O_3_ | 0.35 | 103.055 | 104.10 | 102.89 | 1.00 | Organic Acid |
| 2 | Oxypurinol | C_5_H_4_N_4_O_2_ | 1.15 | 151.015 | 152.11 | 109.04, 121.07, 133.95, 151.95 | 42697.11 | [Nucleotides](https://www.genome.gov/genetics-glossary/Nucleotide) |
| 3 | Uracil | C_4_H_4_N_2_O_2_ | 1.34 | 111.129 | 112.09 | 110.98 | 54.73 | [Nucleotides](https://www.genome.gov/genetics-glossary/Nucleotide) |
| 4 | Mannose 6-phosphate | C_6_H_13_O_9_P | 1.42 | 259.082 | 260.13 | 111.04, 125.04, 241.03, 259.04 | 252.59 | Sugar Phosphate |
| 5 | Gallic acid | C_7_H_6_O_5_ | 1.49 | 168.950 | 170.12 | 109.95, 106.99, 123.00, 150.98 |  | Phenolic Acid |
| 6 | L-Iditol | C_6_H_14_O_6_ | 1.82 | 181.174 | 182.17 | 107.10, 134.04, 163.08 | 37570.48 | Sugar Alcohol |
| 7 | Taurine | C_2_H_7_NO_3_S | 2.07 | 123.869 | 125.15 | 109.02, 105.99, 122.02 | 20646.9 | Amino Acid |
| 8 | N-Carbamoyl-L-Aspartic acid | C_5_H_8_N_2_O_5_ | 2.53 | 174.918 | 176.13 | 100.81, 102.97, 118.98, 130.99, 146.99, 156.91 | 38.51 | Amino Acid |
| 9 | 2-Oxoglutarate | C_5_H_6_O_5_ | 2.62 | 144.919 | 146.10 | 100.80, 116.90 | 19.29 | Organic Acid |
| 10 | Glyceric acid | C_3_H_6_O_4_ | 2.91 | 104.907 | 106.08 | 103.98 | 3.32 | Organic Acid |
| 11 | Citramalate | C_5_H_8_O_5_ | 2.93 | 146.923 | 148.11 | 102.93, 118.94 | 17.42 | Organic Acid |
| 12 | Coumaric acid | C_9_H_8_O_3_ | 3.49 | 162.921 | 164.16 | 100.80, 116.91, 134.96 |  | Phenolic Acid |
| 13 | Inosine | C_10_H_12_N_4_O_5_ | 4.12 | 267.064 | 268.23 | 121.05, 136.07, 151.10, 195.09 | 45.22 | [Nucleotides](https://www.genome.gov/genetics-glossary/Nucleotide) |
| 14 | 6,7-Dihydroxycoumarin | C_9_H_6_O_4_ | 4.82 | 177.020 | 178.14 | 116.99, 149.08, 159.92, 177.00 | 1.36 | Coumarin |
| 15 | Caffeic acid | C_9_H_8_O_4_ | 4.99 | 179.019 | 180.16 | 115.99, 132.96, 150.94, 160.93 | 4.12 | Phenolic Acid |
| 16 | Syringic acid | C_9_H_10_O_5_ | 5.09 | 194.881 | 198.17 | 99.97, 116.69, 159.92, 177.89 | 5.0 e6 | Phenolic Acid |
| 17 | 1,2-Benzenediol | C_6_H_6_O_2_ | 5.18 | 109.138 | 110.11 | 107.97 | 48.92 | Phenol |
| 18 | Ferulic acid | C_10_H_10_O_4_ | 6.10 | 193.011 | 194.19 | 117.07, 134.07, 157.91, 174.95 | 1.7e6 | Phenolic Acid |
| 19 | Magnoflorine | C_20_H_24_NO_4+_ | 6.50 | 341.081 | 342.4 | 109.10, 175.12, 269.15, 283.09, 297.12, 323.18 | 7.8e5 | Alkaloid |
| 20 | (+/-)-cis,trans-abscisic acid | C_15_H_20_O_4_ | 7.07 | 263.142 | 264.32 | 119.03, 129.11, 130.06, 145.08, 161.10, 147.12, 205.11 | 24.96 | Sesquiterpenoid |
| 21 | Hydrastinine | C_11_H_13_NO_3_ | 7.14 | 205.084 | 207.08 | 117.00, 133.03, 175.11 | 3.6e5 | Alkaloid |
| 22 | 3.4-Dihydroxybenzoic acid | C_7_H_6_O_4_ | 7.30 | 152.001 | 154.12 | 109.00, 137.03 |  | Phenolic Acid |
| 23 | Gamma-terpinene | C_10_H_16_ | 7.31 | 135.129 | 136.23 | 100.03, 108.03, 116.92, 134.91 | 3.90 | Monoterpene |
| 24 | Allocryptopine | C_21_H_23_NO_5_ | 7.76 | 369.063 | 369.49 | 116.96, 175.09, 193.11, 245.05, 277.05 | 2.4e6 | Alkaloid |
| 25 | DL-6,8-Thioctic acid | C_8_H_14_O_2_S_2_ | 8.29 | 205.004 | 206.33 | 117.00, 133.10, 143.08, 190.11 | 23362.14 | Organic acid |
| 26 | Guanine | C_5_H_5_N_5_O | 8.39 | 150.137 | 151.13 | 107.01, 122.04 | 19.57 | [Nucleotides](https://www.genome.gov/genetics-glossary/Nucleotide) |
| 27 | Chlorogenic acid | C_16_H_18_O_9_ | 9.15 | 353.052 | 354.31 | 117.00, 145.08, 181.16, 223.16, 247.18, 281.16, 263.13, 335.13 | 9.0e5 | Phenolic Acid |

| **ID** | **Metabolite name** | **Formula** | **RT (min)** | **m/z** | **M.wt** | **Fragments** | **S/N** | **Chemical Class** |
| --- | --- | --- | --- | --- | --- | --- | --- | --- |
| 28 | Canadine | C_20_H_21_NO_4_ | 9.24 | 339.061 | 339.14 | 109.08, 151.15, 163.15, 177.14, 193.14, 267.13, 281.10, 321.13 | 9.8e5 | Alkaloid |
| 29 | Naringenin-7-O-glucoside | C_21_H_22_O_10_ | 9.66 | 433.182 | 434.40 | 137.05, 151.05, 271.15 | 2.61 | Flavonoid |
| 30 | DL-5-Hydroxylysine | C_6_H_14_N_2_O_3_ | 9.88 | 161.153 | 162.19 | 117.00, 145.06 | 30.39 | Amino Acid |
| 31 | Palmatine | C_21_H_22_NO_4+_ | 10.03 | 351.032 | 352.4 | 116.98, 135.14, 189.18, 215.17, 249.24, 289.05, 307.19 | 8.0e5 | Alkaloid |
| 32 | N-Formyl-L-Methionine | C_6_H_11_NO_3_S | 10.29 | 176.172 | 177.22 | 119.04, 133.05, 148.06 | 27.17 | Amino Acid |
| 33 | Berberine | C_20_H_18_NO_4+_ | 10.52 | 333.075 | 336.4 | 137.06, 155.04, 165.15, 189.15, 191.15, 245.23, 289.10 | 1.9e6 | Alkaloid |
| 34 | Farnesol | C_15_H_26_O | 10.77 | 221.229 | 222.37 | 149.13, 206.18 | 32890.74 | Sesquiterpenoid |
| 35 | D-(-)-Arabinose | C_5_H_10_O_5_ | 10.99 | 149.206 | 150.13 | 105.98, 122.01 | 19.98 | Monosaccharide |
| 36 | D-(-)-Quinic acid | C_7_H_12_O_6_ | 13.15 | 191.138 | 192.17 | 133.08, 158.11, 173.15 | 177.50 | Organic Acid |
| 37 | D-(+)-Malic acid | C_4_H_6_O_5_ | 13.85 | 133.174 | 134.09 | 100.78, 115.89 | 47.87 | Organic Acid |
| 38 | Orotic acid | C_5_H_4_N_2_O_4_ | 13.86 | 155.015 | 156.10 | 136.93, 154.95 | 16.09 | [Nucleotides](https://www.genome.gov/genetics-glossary/Nucleotide) |
| 39 | cis-Aconitate | C_6_H_6_O_6_ | 14.28 | 173.022 | 174.11 | 136.89, 154.97 | 1.34 | Organic Acid |
| 40 | 2'-Deoxycytidine | C_9_H_13_N_3_O_4_ | 15.16 | 226.126 | 227.22 | 156.11, 185.17, 210.17 | 10.91 | Deoxynucleoside |
| 41 | Sideroxylin | C_18_H_16_O_5_ | 15.22 | 311.083 | 312.3 | 183.12 | 6.2e6 | Flavonoid |
| 42 | Methylmalonic acid | C_4_H_6_O_4_ | 17.11 | 117.033 | 118.09 | 100.09 | 635.04 | Organic Acid |
| 43 | Resveratrol | C_14_H_12_O_3_ | 19.77 | 227.239 | 228.24 | 111.03, 134.94, 153.09, 199.08, 211.06 | 4.98 | Phenol |
| 44 | Gluconate | C_6_H_12_O_7_ | 21.04 | 194.915 | 196.16 | 99.98, 116.97, 136.15, 159.90, , 177.88 | 766.3 | Sugar Acid |
| 45 | Daidzein | C_15_H_10_O_4_ | 21.13 | 253.215 | 254.24 | 105.00, 121.04, 149.98, 181.07, 234.94 | 7.06 | Flavonoid |
| 46 | Xanthosine | C_10_H_12_N_4_O_6_ | 27.24 | 283.214 | 284.23 | 265.23 | 26.06 | [Nucleotides](https://www.genome.gov/genetics-glossary/Nucleotide) |
| 47 | Hydrastine | C_21_H_21_NO_6_ | 27.30 | 365.242 | 383.4 | 365.28 | 9.6e6 | Alkaloid |
| 48 | Canavanine | C_5_H_12_N_4_O_3_ | 28.37 | 175.074 | 176.17 | 119.02, 138.94, 156.98 | 3.35 | Amino Acid |
| 49 | 3-Hydroxyisovaleric acid | C_5_H_10_O_3_ | 28.45 | 117.101 | 118.13 | 100.71 | 4.71 | Organic Acid |
| 50 | D(-)-Gulono-gamma-lactone | C_6_H_10_O_6_ | 28.63 | 177.036 | 178.14 | 100.42, 116.98, 159.88 | 7.20 | Sugar Lactone |
| 51 | N-alpha-Acetyl-L-Ornithine | C_7_H_14_N_2_O_3_ | 28.80 | 173.095 | 174.20 | 116.86, 136.92, 154.95 | 10.31 | Amino Acid |
| 52 | D-(+)-Galacturonic acid | C_6_H_10_O_7_ | 28.96 | 192.873 | 194.14 | 114.91, 136.92, 157.94, 174.93 | 20429.27 | Sugar Acid |

**Table S5**

Literature and spectral-library references supporting LC–MS/MS-based annotation of metabolites identified in the GS fraction

| **ID** | **Metabolite name** | **Suggested supporting LC-MS/MS reference** | **Reference** |
| --- | --- | --- | --- |
| 1 | HydroxyButyric acid | MassBank **MSBNK-Keio_Univ-KO001011** for **β-hydroxybutyric acid**, LC-ESI-QQ, MS2, [M−H]−. ([massbank.eu](https://massbank.eu/MassBank/RecordDisplay?id=MSBNK-Keio_Univ-KO001011&utm_source=chatgpt.com)) | [4] |
| 2 | Oxypurinol | MassBank **MSBNK-RIKEN-PR100860**, LC-ESI-QTOF, MS2, [M−H]−; additional MassBank oxypurinol MS2 records also exist. ([massbank.eu](https://massbank.eu/MassBank/RecordDisplay?id=MSBNK-RIKEN-PR100860&utm_source=chatgpt.com)) | [5] |
| 3 | Uracil | MassBank **MSBNK-Keio_Univ-KO001977**, LC-ESI-QQ, MS2, [M−H]−. ([massbank.eu](https://massbank.eu/MassBank/RecordDisplay?id=MSBNK-Keio_Univ-KO001977&utm_source=chatgpt.com)) | [6] |
| 4 | Mannose 6-phosphate | MassBank **MSBNK-RIKEN-PR100776**, LC-ESI-QTOF, MS2, [M−H]− for D-mannose-6-phosphate. ([massbank.eu](https://massbank.eu/MassBank/RecordDisplay?id=MSBNK-RIKEN-PR100776&utm_source=chatgpt.com)) | [7] |
| 5 | Gallic acid | PhytoBank negative-mode LC-MS/MS spectrum for gallic acid links to MassBank **KO000888**. ([phytobank.ca](https://phytobank.ca/spectra/ms_ms/6087)) | [8] |
| 6 | L-Iditol | MassBank **MSBNK-RIKEN-PR100483** for L-iditol, LC-ESI-QTOF, MS2, [M−H]−; another L-iditol MS2 record also exists. ([massbank.eu](https://massbank.eu/MassBank/RecordDisplay?id=MSBNK-RIKEN-PR100483&utm_source=chatgpt.com)) | [9] |
| 7 | Taurine | MassBank **MSBNK-NAIST-KNA00481** provides negative-mode taurine MS2 support, though that record uses a precursor at **m/z 249.02** rather than a simple [M−H]− monomer. ([massbank.eu](https://massbank.eu/MassBank/RecordDisplay?id=MSBNK-NAIST-KNA00481&utm_source=chatgpt.com)) | [10] |
| 8 | N-Carbamoyl-L-Aspartic acid | MassBank **MSBNK-Keio_Univ-KO000414** and **KO000415** for carbamoylaspartic acid MS2; a RIKEN record for carbamoyl-DL-aspartic acid also exists. ([massbank.eu](https://massbank.eu/MassBank/RecordDisplay?id=MSBNK-Keio_Univ-KO000414&utm_source=chatgpt.com)) | [11] |
| 9 | 2-Oxoglutarate | MassBank **MSBNK-Keio_Univ-KO001528**, LC-ESI-QQ, MS2, [M−H]−; the record lists precursor m/z 145 and product ions including ~101.1 and 127.0. ([massbank.eu](https://massbank.eu/MassBank/RecordDisplay?id=MSBNK-Keio_Univ-KO001528)) | [12] |
| 10 | Glyceric acid | MassBank **MSBNK-Keio_Univ-KO000861**, LC-ESI-QQ, MS2, [M−H]−. ([massbank.eu](https://massbank.eu/MassBank/RecordDisplay?id=MSBNK-Keio_Univ-KO000861&utm_source=chatgpt.com)) | [13] |
| 11 | Citramalate | MassBank **MSBNK-Metabolon-MT000024**, *Citramalic acid; LC-ESI-IT; MS2; m/z 147.1; [M−H]-*. ([massbank.eu](https://massbank.eu/MassBank/RecordDisplay?id=MSBNK-Metabolon-MT000024&utm_source=chatgpt.com)) | [14] |
| 12 | Coumaric acid | --- | [15] |
| 13 | Inosine | MassBank/CAMP record **MSBNK-NAIST-KNA00710**, *Inosine; LC-ESI-ITFT; MS2; m/z 267.07; NEG*. ([massbank.eu](https://massbank.eu/MassBank/RecordDisplay?id=MSBNK-NAIST-KNA00710&utm_source=chatgpt.com)) | [16] |
| 14 | 6,7-Dihydroxycoumarin (Esculetin) | MassBank has **Esculetin** MS2 records, including **MSBNK-BAFG-CSL23111011231** and **MSBNK-RIKEN_ReSpect-PS125002**. A published LC-MS/MS method for esculetin in **negative ESI** is also available. ([massbank.eu](https://massbank.eu/MassBank/RecordDisplay?id=MSBNK-BAFG-CSL23111011231&utm_source=chatgpt.com)) | [17] |
| 15 | Caffeic acid | MassBank **MSBNK-BGC_Munich-RP016713** (Caffeic acid; LC-ESI-QTOF; MS2; CE 40; **[M−H]−**). | [18] |
| 16 | Syringic acid | MassBank **MSBNK-Keio_Univ-KO001813** (Syringic acid; LC-ESI-QQ; MS2; CE 20 V; **[M−H]−**). | [19] |
| 17 | 1,2-Benzenediol | MassBank **MSBNK-RIKEN-PR100636** (Catechol / 1,2-Benzenediol; LC-ESI-QTOF; MS2; CE 30 V; **[M−H]−**). | [20] |
| 18 | Ferulic acid | MassBank **MSBNK-RIKEN_ReSpect-PM000409**, *Ferulic acid; LC-ESI-QQ; MS2*. DrugBank also mirrors a negative LC-MS/MS spectrum for ferulic acid. ([massbank.eu](https://massbank.eu/MassBank/RecordDisplay?id=MSBNK-RIKEN_ReSpect-PM000409&utm_source=chatgpt.com)) | [21] |
| 19 | Magnoflorine | --- | [22] |
| 20 | (+/-)-cis,trans-Abscisic acid | MassBank **MSBNK-Washington_State_Univ-BML80671**, *Abscisic acid; LC-ESI-QTOF; MS; NEGATIVE*, precursor at **m/z 263.1289**. Published LC-ESI-MS/MS methods for ABA in negative mode also exist. ([massbank.eu](https://massbank.eu/MassBank/RecordDisplay?id=MSBNK-Washington_State_Univ-BML80671&utm_source=chatgpt.com)) | [23] |
| 21 | Hydrastinine | --- | [24] |
| 22 | 3,4-Dihydroxybenzoic acid (Protocatechuic acid) | MassBank **MSBNK-RIKEN_ReSpect-PT203280**, **3,4-dihydroxybenzoic acid; LC-ESI-QTOF; MS2**. ([massbank.eu](https://massbank.eu/MassBank/RecordDisplay?id=MSBNK-RIKEN_ReSpect-PT203280&utm_source=chatgpt.com)) | [25] |
| 23 | Gamma-terpinene | The MassBank record I found for **gamma-terpinene** is an **MS** spectrum, not LC–ESI-MS/MS fragmentation, so it is weak support for your reviewer response. ([massbank.eu](https://massbank.eu/MassBank/RecordDisplay?id=MSBNK-Fac_Eng_Univ_Tokyo-JP007396&utm_source=chatgpt.com)) | [26] |
| 24 | Allocryptopine |  | [27] |
| 25 | DL-6,8-Thioctic acid (DL-Thioctic acid) | MassBank has **DL-Thioctic acid** records in **negative mode**, including **LC-APCI-QTOF; MS; NEGATIVE** and an **MS2** record. ([massbank.eu](https://massbank.eu/MassBank/RecordDisplay?id=MSBNK-Washington_State_Univ-BML81143&utm_source=chatgpt.com)) | [28] |
| 26 | Guanine | --- | [29] |
| 27 | Chlorogenic acid | MassBank **MSBNK-Keio_Univ-KO000469**, **Chlorogenic acid; LC-ESI-QQ; MS2; CE 40 V; [M−H]−**. ([massbank.eu](https://massbank.eu/MassBank/RecordDisplay?id=MSBNK-Keio_Univ-KO000469&utm_source=chatgpt.com)) | [30] |
| 28 | Canadine | --- | [31] |
| 29 | Naringenin-7-O-glucoside | MassBank **MSBNK-RIKEN-PR309316** (Naringenin-7-O-glucoside; LC-ESI-QTOF; MS2). ([MassBank](https://massbank.eu/MassBank/RecordDisplay?id=MSBNK-RIKEN-PR309316&utm_source=chatgpt.com)) | [32] |
| 30 | DL-5-Hydroxylysine | --- | [33] |
| 31 | Palmatine | --- | [34] |
| 32 | N-Formyl-L-Methionine | --- | [35] |
| 33 | Berberine | --- | [36] |
| 34 | Farnesol | --- | [37] |
| 35 | D-(-)-Arabinose | --- | [38] |
| 36 | D-(-)-Quinic acid | MassBank **MSBNK-Keio_Univ-KO001746** (Quinic acid; LC-ESI-QQ; MS2; CE 20 V; **[M−H]−**). ([MassBank](https://massbank.eu/MassBank/RecordDisplay?id=MSBNK-Keio_Univ-KO001746&utm_source=chatgpt.com" \o "Quinic acid; LC-ESI-QQ; MS2; CE:20 V; [M-H]- - MassBank)) | [39] |
| 37 | D-(+)-Malic acid | MassBank **MSBNK-Keio_Univ-KO001307** (Malic acid; LC-ESI-QQ; MS2; CE 30 V; **[M−H]−**). ([MassBank](https://massbank.eu/MassBank/RecordDisplay?id=MSBNK-Keio_Univ-KO001307&utm_source=chatgpt.com" \o "Malic acid; LC-ESI-QQ; MS2; CE:30 V; [M-H]- - MassBank)) | [40] |
| 38 | Orotic acid | MassBank **MSBNK-RIKEN-PR100595** (Orotic acid; LC-ESI-QTOF; MS2; **[M−H]−**). ([MassBank](https://massbank.eu/MassBank/RecordDisplay?id=MSBNK-RIKEN-PR100595&utm_source=chatgpt.com" \o "Orotic acid; LC-ESI-QTOF; MS2; CE:Ramp 5-60 V; [M-H]- - MassBank)) | [41] |
| 39 | cis-Aconitate | MassBank **MSBNK-RIKEN-PR100734** (cis-Aconitic acid; LC-ESI-QTOF; MS2; **[M−H]−**). ([MassBank](https://massbank.eu/MassBank/RecordDisplay?id=MSBNK-RIKEN-PR100734&utm_source=chatgpt.com" \o "cis-Aconitic acid; LC-ESI-QTOF; MS2; CE:Ramp 5-60 V; [M-H]- - MassBank)) | [42] |
| 40 | 2'-Deoxycytidine | MassBank **MSBNK-RIKEN-PR100522** (2'-Deoxycytidine; LC-ESI-QTOF; MS2; **[M−H]−**). ([MassBank](https://massbank.eu/MassBank/RecordDisplay?id=MSBNK-RIKEN-PR100522&utm_source=chatgpt.com" \o "2'-Deoxycytidine; LC-ESI-QTOF; MS2; CE:Ramp 5-60 V; [M-H]- - MassBank)) | [43] |
| 41 | Sideroxylin | --- | [44] |
| 42 | Methylmalonic acid | MassBank **MSBNK-Keio_Univ-KO001342** (Methylmalonic acid; LC-ESI-QQ; MS2; CE 50 V; **[M−H]−**). ([MassBank](https://massbank.eu/MassBank/RecordDisplay?id=MSBNK-Keio_Univ-KO001342&utm_source=chatgpt.com" \o "Methylmalonic acid; LC-ESI-QQ; MS2; CE:50 V; [M-H]- - MassBank)) | [45] |
| 43 | Resveratrol | --- | [46] |
| 44 | Gluconate | --- | [47] |
| 45 | Daidzein | MassBank **MSBNK-BAFG-CSL2311092159** (Daidzein; LC-ESI-QTOF; MS2). A separate MassBank record for daidzein also exists. ([MassBank](https://massbank.eu/MassBank/RecordDisplay?id=MSBNK-BAFG-CSL2311092159&utm_source=chatgpt.com)) | [48] |
| 46 | Xanthosine | MassBank **MSBNK-RIKEN-PR100731** (Xanthosine; LC-ESI-QTOF; MS2; **[M−H]−**). ([MassBank](https://massbank.eu/MassBank/RecordDisplay?id=MSBNK-RIKEN-PR100731&utm_source=chatgpt.com" \o "Xanthosine; LC-ESI-QTOF; MS2; CE:Ramp 5-60 V; [M-H]- - MassBank)) | [49] |
| 47 | Hydrastine | --- | [50] |
| 48 | Canavanine | --- | [51] |
| 49 | 3-Hydroxyisovaleric acid | --- | [52] |
| 50 | D(-)-Gulono-gamma-lactone | --- | [53] |
| 51 | N-alpha-Acetyl-L-Ornithine | --- | [54] |
| 52 | D-(+)-Galacturonic acid | MassBank **MSBNK-RIKEN-PR100504** (D-(+)-Galacturonic acid; LC-ESI-QTOF; MS2; **[M−H]−**). ([MassBank](https://massbank.eu/MassBank/RecordDisplay?id=MSBNK-RIKEN-PR100504&utm_source=chatgpt.com" \o "D- (+)-Galacturonic acid; LC-ESI-QTOF; MS2; CE:Ramp 5-60 V; [M-H]-)) | [55] |

--- No verified compound-specific negative-mode LC-MS/MS

**Table S6**

The binding free energy (∆G) for the group of fractioned compounds that were screened using the target protein receptors *RhIR, Rh1I, LasR, and LasI.*

| **Numbers and names of chemical fractions** | ***RhIR***  **Kcal / mol** | ***Rh1I***  **Kcal / mol** | ***LasR***  **Kcal / mol** | ***LasI***  **Kcal / mol** |
| --- | --- | --- | --- | --- |
| 1-HydroxyButyric acid | -2.79 | -3.17 | -3.18 | -2.973 |
| 2-Oxypurinol | -5.76 | -6.57 | -6.78 | -6.20 |
| 3-Uracil | -5.56 | -6.33 | -7.19 | -5.87 |
| 4-Mannose 6-phosphate | -7.76 | -6.64 | -7.76 | -5.99 |
| 5-Gallic acid | -6.24 | -6.44 | -7.17 | -6.28 |
| 6-L-Iditol | -4.29 | -5.00 | -5.53 | -3.66 |
| 7-Taurine | -5.03 | -4.70 | -5.38 | -5.07 |
| 8-N-Carbamoyl-L-Aspartic acid | -6.43 | -5.50 | -6.74 | -5.08 |
| 9-2-oxopentanedioic acid | -4.51 | -4.55 | -4.71 | -4.08 |
| 10-Glyceric acid | -4.10 | -4.03 | -3.67 | -4.00 |
| 11-Citramalate | -5.68 | -5.15 | -4.69 | -4.74 |
| 12-p-Coumaric acid | -5.91 | -5.87 | -7.13 | -4.46 |
| 13-Inosine | -7.24 | -6.49 | -7.96 | -6.04 |
| 14-6,7-Dihydroxycoumarin | -6.70 | -6.46 | -7.14 | -4.91 |
| 15-Caffeic acid | -6.15 | -5.93 | -6.55 | -4.86 |
| 16-Syringic acid | -6.67 | -6.54 | -6.71 | -6.12 |
| 17-1,2-Benzenediol | -6.31 | -4.97 | -7.07 | -4.80 |
| 18-Ferulic acid | -6.55 | -6.32 | -7.47 | -4.77 |
| 19-Magnoflorine | -6.96 | -6.72 | -6.36 | -4.62 |
| 20-cis,trans-abscisic acid | -7.00 | -6.76 | -7.98 | -4.26 |
| 21-Hydrastinine | -7.66 | -7.16 | -8.09 | -5.29 |
| 22-3.4-Dihydroxybenzoic acid | -6.00 | -6.01 | -6.98 | -5.91 |
| 23-Gamma-terpinene | -5.80 | -5.43 | -7.09 | -4.60 |
| 24-Allocryptopine | -7.66 | -6.84 | -5.54 | -4.05 |
| 25-DL-6,8-Thioctic acid | -5.19 | -5.47 | -6.63 | -4.91 |
| 26-Guanine | -6.37 | -7.28 | -7.12 | -6.19 |
| 27-Chlorogenic acid | -7.85 | -7.98 | -7.83 | -6.30 |
| 28-Canadine | -7.37 | -6.97 | -7.25 | -4.01 |
| 29-Naringenin-7-O-glucoside | -8.47 | -7.30 | -9.38 | -5.97 |
| 30-DL-5-Hydroxylysine | -5.63 | -4.66 | -5.66 | -4.55 |
| 31-Palmatine | -7.40 | -6.94 | -7.11 | -4.95 |
| 32-N-Formyl-L-Methionine | -6.66 | -4.92 | -4.53 | -3.81 |
| 33-Berberine | -6.49 | -7.23 | -7.96 | -4.65 |
| 34-Farnesol | -3.30 | -3.72 | -3.07 | -1.66 |
| 35-D-(-)-Arabinose | -4.21 | -4.08 | -5.41 | -4.38 |
| 36-D-(-)-Quinic acid | -6.17 | -7.00 | -6.44 | -5.31 |
| 37-D-(+)-Malic acid | -4.64 | -4.56 | -4.86 | -4.61 |
| 38-Orotic acid | -6.17 | -7.51 | -7.12 | -7.42 |
| 39-cis-Aconitate | -6.25 | -5.66 | -6.04 | -4.86 |
| 40-2'-Deoxycytidine | -7.30 | -6.58 | -6.89 | -5.15 |
| 41-Sideroxylin | -7.41 | -7.01 | -6.92 | -4.66 |
| 42-Methylmalonic acid | -4.71 | -4.02 | -4.33 | -4.51 |
| 43-Resveratrol | -7.70 | -7.01 | -6.55 | -4.80 |
| 44-Gluconate | -4.46 | -5.05 | -5.91 | -5.57 |
| 45-Daidzein | -8.47 | -7.38 | -7.84 | -5.69 |
| 46-Xanthosine | -7.29 | -6.67 | -7.84 | -5.73 |
| 47-Hydrastine | -7.23 | -6.76 | -6.78 | -4.27 |
| 48-Canavanine | -1.59 | -1.01 | -2.12 | -2.41 |
| 49-3-Hydroxyisovaleric acid | -4.74 | -3.88 | -3.78 | -4.26 |
| 50-D(-)-Gulono-gamma-lactone | -5.55 | -5.19 | -6.05 | -4.39 |
| 51-N-alpha-Acetyl-L-Ornithine | -5.20 | -5.30 | -5.50 | -4.18 |
| 52-D-(+)-Galacturonic acid | -6.07 | -6.40 | -7.40 | -7.06 |

**Table S7**

Best binding compound per receptor

| **Receptor** | **Compound (No. and Name)** | **Binding affinity (kcal/mol)** |
| --- | --- | --- |
| *LasR* | 29 – Naringenin-7-O-glucoside | –9.38 |
| *RhIR* | 29 – Naringenin-7-O-glucoside / 45 – Daidzein | –8.47 |
| *Rh1I* | 27 – Chlorogenic acid | –7.98 |
| *LasI* | 38 – Orotic acid | –7.42 |

**Table S8**

Screening of antibiotic activity and susceptibility of the isolates

| **Antibiotic** | **Resistant** | | **Intermediate** | | **Sensitive** | |
| --- | --- | --- | --- | --- | --- | --- |
|  | **No.** | **%** | **No.** | **%** | **No.** | **%** |
| Ticarcillin | 100 | 100% | 0 | 0% | 0 | 0% |
| Ticarcillin/clavulanic acid | 100 | 100% | 0 | 0% | 0 | 0% |
| Piperacillin | 100 | 100% | 0 | 0% | 17 | 17% |
| Piperacillin/tazobactam | 80 | 80% | 0 | 0% | 20 | 20% |
| Ceftazidime | 50 | 50% | 0 | 0% | 50 | 50% |
| Cefepime | 45 | 50% | 5 | 5% | 50 | 50% |
| Imipenem | 40 | 40% | 10 | 10% | 50 | 50% |
| amikacin | 30 | 30% | 10 | 10% | 60 | 60% |
| Ciprofloxacin | 30 | 30% | 0 | 0% | 70 | 70% |
| Colistin | 0 | 0% | 0 | 0% | 100 | 100% |

**Table S9**

The percentage of biofilm production in *P. aeruginosa* isolates

| **Biofilm degree** | **No (%)** |
| --- | --- |
| High producer | 60(60%) |
| Intermediate producer | 20(20%) |
| Low producer | 20(20%) |
| No Biofilm production | 0(0%) |

**Table S10**

Relative Gene Expression (Fold Change and CT Values) of rhlR, rhlI, lasR, and lasI Normalized to 16S rDNA in control and sample

| ***rhlR*** | | ***rhlI*** | | ***lasR*** | | ***lasI*** | | ***16S rDNA*** | **Sample**  **No.** |
| --- | --- | --- | --- | --- | --- | --- | --- | --- | --- |
| **Fold change** | **CT** | **Fold change** | **CT** | **Fold change** | **CT** | **Fold change** | **CT** |  |  |
|  |  |  |  |  |  |  |  | **CT** |  |
| **-** | 21.61 | **-** | 20.89 | **-** | 21.46 | **-** | 22.16 | 19.52 | A |
| **0.1539** | 24.60 | **0.4796** | 22.24 | **0.0461** | 26.19 | **0.1604** | 25.09 | 19.81 | B |

**Table S11**

Normalized effect scores and statistical comparison between topical and oral GS treatments

| **Parameter** | **Direction** | **Oral GS** | **Topical GS** | **p-value (Topical vs Oral)** |
| --- | --- | --- | --- | --- |
| TNF-α | ↓ | 0.43 | 0.79 | < 0.001 |
| IL-1β | ↓ | 0.32 | 0.78 | < 0.001 |
| CAMP | ↑ | 0.62 | 1.00 | < 0.001 |
| RBD2 | ↑ | 0.50 | 1.00 | < 0.001 |
| VEGF | ↑ | 0.67 | 1.00 | < 0.001 |
| bFGF | ↑ | 0.65 | 1.00 | < 0.001 |

Normalized effect scores (0–1) were derived from mean values presented in Figs. 8 and 9. For inflammatory markers (TNF-α, IL-1β), higher scores indicate greater reduction; for regenerative markers (CAMP, RBD2, VEGF, bFGF), higher scores indicate greater upregulation. Data are based on n = 6 animals per group. Statistical comparisons were performed between topical and oral GS treatments.

**Fig. S1** LC-ESI-MS/MS chromatograms obtained in MRM mode of *goldenseal* fraction.

**Fig. S2**	Effect of *GS* bioactive fraction on *P. aeruginosa* clinically isolates.

**Fig. S3** DNA extraction and PCR amplification methodology to detect the presence of quorum sensing genes *LasR, LasI, RhIR, RhII.*

**Fig. S4** Influence of *GS* bioactive fraction on *lasR , lasI, rhII, and rhlR* genes expression levels.

**Fig. S5**

Dissociation (melting) curves obtained from qPCR analysis of 16S rDNA (reference gene) and quorum-sensing genes (*lasR, lasI, rhlR,* and *rhlI*). Each panel represents the melting profile of a specific gene, showing fluorescence change as a function of temperature. The presence of a single sharp peak in each curve indicates specific amplification without nonspecific products or primer-dimer formation.

**References**

1. Spilker, T., Coenye, T., Vandamme, P. & LiPuma, J. J. PCR-based assay for differentiation of *Pseudomonas aeruginosa* from other *Pseudomonas* species recovered from cystic fibrosis patients. *J. Clin. Microbiol.* 42, 2074–2079 (2004).
2. Bratu, S., Gupta, J. & Quale, J. Expression of the *las* and *rhl* quorum-sensing systems in clinical isolates of *Pseudomonas aeruginosa* does not correlate with efflux pump expression or antimicrobial resistance. *J. Antimicrob. Chemother.* 58, 1250–1253 (2006).
3. Saleh, M. M., Sadeq, R. A., Latif, H. K. A., Abbas, H. A. & Askoura, M. Zinc oxide nanoparticles inhibit quorum sensing and virulence in *Pseudomonas aeruginosa*. *Afr. Health Sci.* 19, 2043–2055 (2019).

4. Kim, S., Lee, M. S., Kim, M., Ko, B. J., Lee, H. S. & Lee, S. Derivatization-assisted LC-MS/MS method for simultaneous quantification of endogenous gamma-hydroxybutyric acid and its metabolic precursors and products in human urine. *Anal. Chim. Acta* 1194, 339401 (2022).

5. Kasawar, G., Razzak, M., Zaheer, Z. & Farooqui, M. Validated RP-LC-MS/MS method for the simultaneous determination of allopurinol and its major metabolite, oxypurinol, in human plasma. *J. Liq. Chromatogr. Relat. Technol.* 34, 26–37 (2010).

6. Büchel, B., Rhyn, P., Schürch, S., Bühr, C., Amstutz, U. & Largiadèr, C. R. LC-MS/MS method for simultaneous analysis of uracil, 5,6-dihydrouracil, 5-fluorouracil and 5-fluoro-5,6-dihydrouracil in human plasma for therapeutic drug monitoring and toxicity prediction in cancer patients. *Biomed. Chromatogr.* 27, 7–16 (2013).

7. Sleat, D. E., Della Valle, M. C., Zheng, H., Moore, D. F. & Lobel, P. The mannose 6-phosphate glycoprotein proteome. *J. Proteome Res.* 7, 3010–3021 (2008).

8. Hamiti, X., Shallari, G., Pupuleku, B., Yücel, A., Çelik, S., Sulejmani, E. & Lazo, P. Phenolic profiling of Albanian honeys by LC-MS/MS: gallic acid as a predictive marker of antioxidant potential. *Molecules* 30, 4037 (2025).

9. Wong, Y. T., Or, M. S., Lau, H. H., Wong, S. C. & Fung, W. H. Analysis of underivatized alditols in traditional Chinese medicines by liquid chromatography–tandem mass spectrometry based on ligand exchange chromatography. *Sep. Sci. Plus* 4, 6–15 (2021).

10. Seon, Y. J., Seo, H. J., Yoon, J., Cho, H., Hong, S., Lee, S. H. & Na, T. W. Advanced analytical method through quantitative comparative study of taurine in feed using LC-MS/MS. *Mass Spectrom. Lett.* 13, 133–138 (2022).

11. Sang, Y., Ren, K., Chen, Y., Wang, B., Meng, Y., Zhou, W., Jiang, Y. & Xu, J. Integration of soil microbiology and metabolomics to elucidate the mechanism of accelerated infestation of tobacco by the root-knot nematode. *Front. Microbiol.* 15, 1455880 (2024).

12. Oldham, W. M. & Loscalzo, J. Quantification of 2-hydroxyglutarate enantiomers by liquid chromatography-mass spectrometry. *Bio Protoc.* 6, e1908 (2016).

13. Della Corte, A., Chitarrini, G., Di Gangi, I. M., Masuero, D., Soini, E., Mattivi, F. & Vrhovsek, U. A rapid LC-MS/MS method for quantitative profiling of fatty acids, sterols, glycerolipids, glycerophospholipids and sphingolipids in grapes. *Talanta* 140, 52–61 (2015).

14. Zaslaver, O. Metabolomics analysis reveals novel aspects of 2-hydroxyglutarate homeostasis and links citramalate to branched chain amino acid metabolism. *Univ. Toronto* (2018).

15. Culhaoglu, T., Zheng, D., Méchin, V. & Baumberger, S. Adaptation of the Carrez procedure for the purification of ferulic and p-coumaric acids released from lignocellulosic biomass prior to LC/MS analysis. *J. Chromatogr. B* 879, 3017–3022 (2011).

16. Jimmerson, L. C., Bushman, L. R., Ray, M. L., Anderson, P. L. & Kiser, J. J. LC-MS/MS method for quantifying adenosine, guanosine and inosine nucleotides in human cells. *Pharm. Res.* 34, 73–83 (2017).

17. Yun, E. S., Park, S. K., Kim, B. S., Chae, Y. Z., Cho, S. M., Yi, H., Cho, H. J. & Shin, H. C. Determination of esculetin contents of medicinal plants by LC-MS/MS. *Biomed. Chromatogr.* 26, 1247–1251 (2012).

18. Wang, X., Li, W., Ma, X., Chu, Y., Li, S., Guo, J., Jia, Y., Zhou, S., Zhu, Y. & Liu, C. Simultaneous determination of caffeic acid and its major pharmacologically active metabolites in rat plasma by LC-MS/MS and application in pharmacokinetic study. *Biomed. Chromatogr.* 29, 552–559 (2015).

19. Bursal, E., Köksal, E., Gülçin, İ., Bilsel, G. & Gören, A. C. Antioxidant activity and polyphenol content of cherry stem (Cerasus avium L.) determined by LC-MS/MS. *Food Res. Int.* 51, 66–74 (2013).

20. Draper, W. M., Wijekoon, D., McKinney, M., Behniwal, P., Perera, S. K. & Flessel, C. P. Atmospheric pressure ionization LC-MS-MS determination of urushiol congeners. *J. Agric. Food Chem.* 50, 1852–1858 (2002).
21. Jong, T. T., Lee, M. R., Chiang, Y. C. & Chiang, S. T. Using LC/MS/MS to determine matrine, oxymatrine, ferulic acid, mangiferin, and glycyrrhizin in Chinese medicinal preparations. *J. Pharm. Biomed. Anal.* 40, 472–477 (2006).
22. Tian, X., Li, Z., Lin, Y., Chen, M., Pan, G. & Huang, C. Study on pharmacokinetic profiles of magnoflorine and its interaction in Cortex phellodendri decoction by LC-MS/MS. *Anal. Bioanal. Chem.* 406, 841–849 (2014).
23. Alipour, M., Haghighi, M., Rahimmalek, M., Reichelt, M., Martinelli, L., Groten, K. & Mithöfer, A. Integrated metabolomics, transcriptomic and phytohormonal analyses of water stress and abscisic acid effects in *Thymus* species using LC-MS/MS. *Front. Plant Sci.* 16, 1557446 (2025).
24. Pillai, M. G., Kumar, A., Sharma, R. & Bhasin, N. LC–MS based workflows for qualitative and quantitative analysis of homeopathic preparation of *Hydrastis canadensis*. *Chromatographia* 77, 119–131 (2014).
25. Ma, F., Gong, X., Zhou, X., Zhao, Y. & Li, M. UHPLC–MS/MS method for simultaneous quantification of gallic acid and protocatechuic acid in rat plasma and its pharmacokinetic application. *J. Ethnopharmacol.* 162, 377–383 (2015).
26. Grevsen, K., Fretté, X. C. & Christensen, L. P. Content and composition of volatile terpenes, flavonoids and phenolic acids in Greek oregano (*Origanum vulgare* L. ssp. *hirtum*) during cultivation. *Eur. J. Hortic. Sci.* 74, 193 (2009).
27. Song, K., Oh, J. H., Lee, M. Y., Lee, S. G. & Ha, I. J. Molecular network-guided alkaloid profiling of aerial parts of *Papaver nudicaule* L. using LC-HRMS. *Molecules* 25, 2636 (2020).
28. Wei, Z., Jeong, H. C., Kang, Y. J., Jang, J., Kim, M. H. & Shin, K. H. Bioanalytical methods for detection of duloxetine and thioctic acid in plasma using UPLC-MS/MS. *Transl. Clin. Pharmacol.* 30, 99 (2022).
29. Shih, Y. M., Cooke, M. S., Pan, C. H., Chao, M. R. & Hu, C. W. Clinical relevance of guanine-derived urinary biomarkers of oxidative stress determined by LC-MS/MS. *Redox Biol.* 20, 556–565 (2019).
30. Clifford, M. N., Wu, W., Kirkpatrick, J. & Kuhnert, N. Profiling chlorogenic acids and other caffeic acid derivatives of herbal *Chrysanthemum* by LC–MSⁿ. *J. Agric. Food Chem.* 55, 929–936 (2007).
31. Patel, D. K. Medicinal importance, pharmacological activities and analytical aspects of canadine in medicine. *Curr. Chin. Chem.* 2, e230921196750 (2022).
32. Aghakhani, F., Kharazian, N. & Lori Gooini, Z. Flavonoid constituents of *Phlomis* species using liquid chromatography–mass spectrometry. *Phytochem. Anal.* 29, 180–195 (2018). Bottom of Form
33. Shan, M., Liu, H., Hao, Y., Song, K., Meng, T., Feng, C., Wang, Y. & Huang, Y. Metabolomic profiling reveals that 5-hydroxylysine and 1-methylnicotinamide are metabolic indicators of keloid severity. *Front. Genet.* 12, 804248 (2022).
34. Yang, Q. C., Wu, W. H., Han, F. M. & Chen, Y. Identification of in vivo and in vitro metabolites of palmatine by liquid chromatography–tandem mass spectrometry. *J. Pharm. Pharmacol.* 61, 647–652 (2009).
35. Xia, C., Tian, Q., Kong, L., Sun, X., Shi, J., Zeng, X. & Pan, D. Metabolomics analysis for nitrite degradation by metabolites of *Limosilactobacillus fermentum* RC4. *Foods* 11, 1009 (2022).
36. Naaz, I. & Ali, S. A. Identification and characterization of bioactive compound berberine in *Berberis vulgaris* root extract using HR-LC-MS analysis. *J. Anal. Pharm. Res.* 7, 146–150 (2018).
37. Moinuddin, S. K., Gajbhiye, R. L., Mehta, P., Sarmah, B., Murty, U. S., Ravichandiran, V., Samudrala, P. K., Alexander, A. & Kumar, P. UHPLC-DAD method development and validation: degradation kinetics, stress studies of farnesol and characterization of degradation products using LC-QTOF-ESI-MS with in silico predictions. *J. Chromatogr. Sci.* 60, 817–831 (2022).
38. Liu, Y., Muema, F. W., Zhang, Y. L. & Guo, M. Q. Acyl quinic acid derivatives screened from *Carissa spinarum* by SOD-affinity ultrafiltration LC–MS and their antioxidative and hepatoprotective activities. *Antioxidants* 10, 1302 (2021).
39. Fang, N., Yu, S. & Prior, R. L. LC/MS/MS characterization of phenolic constituents in dried plums. *J. Agric. Food Chem.* 50, 3579–3585 (2002).
40. Fernández-Fernández, R., López-Martínez, J. C., Romero-González, R., Martínez-Vidal, J. L., Alarcón Flores, M. I. & Garrido Frenich, A. Simple LC–MS determination of citric and malic acids in fruits and vegetables. *Chromatographia* 72, 55–62 (2010).
41. Rashed, M. S., Jacob, M., Al-Amoudi, M., Rahbeeni, Z., Al-Sayed, M. A., Al-Ahaidib, L., Saadallah, A. A. & Legaspi, S. Rapid determination of orotic acid in urine by LC–electrospray tandem mass spectrometry. *Clin. Chem.* 49, 499–501 (2003).
42. Jaitz, L., Mueller, B., Koellensperger, G., Huber, D., Oburger, E., Puschenreiter, M. & Hann, S. LC–MS analysis of low molecular weight organic acids derived from root exudation. *Anal. Bioanal. Chem.* 400, 2587–2596 (2011).
43. Richardson, F. C., Zhang, C., Lehrman, S. R., Koc, H., Swenberg, J. A., Richardson, K. A. & Bendele, R. A. Quantification of 2′-fluoro-2′-deoxyuridine and 2′-fluoro-2′-deoxycytidine in DNA and RNA using LC/MS/MS. *Chem. Res. Toxicol.* 15, 922–926 (2002).
44. Junio, H. A. Goldenseal: A case study on LC-MS profiling to identify synergists. *Univ. North Carolina Greensboro* (2011).
45. Zhang, C., Bai, N., Yang, Y., Cheng, Y., He, X., Wang, M., Zhou, H. & Tian, Y. Reliable LC-MS/MS method development and validation for determination of organic acids and homocysteine in dried blood spots. *Clin. Biochem.* 111013 (2025).
46. Lyons, M. M., Yu, C., Toma, R. B., Cho, S. Y., Reiboldt, W., Lee, J. & van Breemen, R. B. Resveratrol in raw and baked blueberries and bilberries. *J. Agric. Food Chem.* 51, 5867–5870 (2003).
47. Park, Y. J., Sim, S., Jung, S., Seo, H., Lee, Y., Lee, J., Lee, C. & Suh, H. J. Simultaneous quantification of ferrous gluconate and calcium gluconate in foods using LC–MS/MS. *Appl. Biol. Chem.* 66, 31 (2023).
48. Erdemoglu, N., Tosun, F. & Eroglu, Y. LC-MS analysis of daidzein in Turkish *Genista* species. *Chem. Nat. Compd.* 42, 517–519 (2006).
49. Tao, N., Wang, R., Xu, X., Dong, T., Zhang, S., Liang, M. & Wang, Q. Xanthosine as a novel anti-browning compound in potato identified by metabolomic analysis. *Postharvest Biol. Technol.* 171, 111367 (2021).
50. Le, P. M., McCooeye, M. & Windust, A. Characterization of alkaloids in goldenseal (*Hydrastis canadensis*) root by high-resolution Orbitrap LC-MSn. *Anal. Bioanal. Chem.* 405, 4487–4498 (2013).
51. Krakauer, J., Long, Y., Kolbert, A., Thanedar, S. & Southard, J. Presence of L-canavanine in *Hedysarum alpinum* seeds and its potential biological implications. *Wilderness Environ. Med.* 26, 36–42 (2015).
52. Nagatomo, R., Kaneko, H., Kamatsuki, S., Ichimura-Shimizu, M., Ishimaru, N., Tsuneyama, K. & Inoue, K. Short-chain fatty acid profiling in a mouse model of Sjögren’s syndrome using derivatized LC-MS/MS. *J. Chromatogr. B* 1210, 123432 (2022).
53. Gao, H., Gong, L., Ni, J. & Li, Q. Metabolomics analysis of lettuce (*Lactuca sativa* L.) under low potassium supply. *Agriculture* 12, 1153 (2022).
54. Martens-Lobenhoffer, J., Becker, A., Freude, H. & Bode-Böger, S. M. Identification and quantification of ornithine-lactam in human plasma by LC–MS/MS. *J. Chromatogr. B* 877, 2284–2289 (2009).
55. Li, Y., Liang, J., Gao, J. N., Shen, Y., Kuang, H. X. & Xia, Y. G. Novel LC-MS/MS method for complete composition analysis of polysaccharides using aldononitrile acetate and MRM. *Carbohydr. Polym.* 272, 118478 (2021). Top of Form

Bottom of Form
